# Supplementary material for: Microwave-Assisted Synthesis of 3-Hydroxy-2-oxindoles and Pilot Evaluation of Their Antiglaucomic Activity
Source: Int J Mol Sci. 2023 Mar 7;24(6):5101. doi: 10.3390/ijms24065101 (PMC10049166; doi:10.3390/ijms24065101)

Supplementary Materials

Microwave-Assisted Synthesis of 3-hydroxy-2-oxindoles and Pilot Evaluation of Their Antiglaucomic Activity

Alexander M. Efremov[1,2], Olga V. Beznos[3], Roman O. Eremeev[1], Natalia B. Chesnokova[3], Elena R. Milaeva[1,2], Elena F. Shevtsova[2] and Natalia A. Lozinskaya[1,*]

1 Department of Chemistry, Lomonosov Moscow State University, Moscow 119991, Russia
2 Institute of Physiologically Active Compounds at Federal Research Center of Problems of Chemical Physics and Medicinal Chemistry, Russian Academy of Sciences (IPAC RAS), Chernogolovka 142432, Russia
3 Helmholtz National Medical Center of Eye Diseases, 14/19 Sadovaya-Chernogryazskaya St., Moscow 105062, Russia
* Correspondence: natalylozinskaya@mail.ru

table of contents

S1. 1H NMR spectrum of (3-hydroxy-5-nitro-2-oxo-2,3-dihydro-1H-indol-3-yl)acetonitrile (**2a**) 3

S2. 1H NMR spectrum of (3-hydroxy-7-nitro-2-oxo-2,3-dihydro-1H-indol-3-yl)acetonitrile (2b) 4

S3. 1H NMR spectrum of (3-hydroxy-5-methoxy-2-oxo-2,3-dihydro-1H-indol-3-yl)acetonitrile (**2c**) 5

S4. 13C NMR spectrum of (3-hydroxy-5-methoxy-2-oxo-2,3-dihydro-1H-indol-3-yl)acetonitrile (**2c**) 6

S5. 1H NMR spectrum of (3-hydroxy-5-methoxy-4-nitro-2-oxo-2,3-dihydro-1H-indol-3-yl)acetonitrile (**2d**) 7

S6. 1H NMR spectrum of (3-hydroxy-5-methoxy-7-nitro-2-oxo-2,3-dihydro-1H-indol-3-yl)acetonitrile (**2e**) 8

S7. 13C NMR spectrum of (3-hydroxy-5-methoxy-7-nitro-2-oxo-2,3-dihydro-1H-indol-3-yl)acetonitrile (**2e**) 9

S8. 1H NMR spectrum of (3-hydroxy-5,7-dinitro-2-oxo-2,3-dihydro-1H-indol-3-yl)acetonitrile (**2f**) 10

S9. 1H NMR spectrum of (3-hydroxy-2-oxo-2,3-dihydro-1H-indol-3-yl)acetonitrile (**2g**) 11

S10. 1H NMR spectrum of (5-bromo-3-hydroxy-2-oxo-2,3-dihydro-1H-indol-3-yl)acetonitrile (**2h**) 12

S11. 13C NMR spectrum of (5-bromo-3-hydroxy-2-oxo-2,3-dihydro-1H-indol-3-yl)acetonitrile (**2h**) 13

S12. 1H NMR spectrum of (1-benzyl-3-hydroxy-5-methoxy-2-oxo-2,3-dihydro-1H-indol-3-yl)acetonitrile (**2i**) 14

S13. 13C NMR spectrum of (1-benzyl-3-hydroxy-5-methoxy-2-oxo-2,3-dihydro-1H-indol-3-yl)acetonitrile (**2i**) 15
S14. 1H NMR spectrum of (3-hydroxy-5-nitro-2-oxo-2,3-dihydro-1H-indol-3-yl)acetic acid (**3a**) 16
S15. 1H NMR spectrum of (3-hydroxy-7-nitro-2-oxo-2,3-dihydro-1H-indol-3-yl)acetic acid (**3b**) 17
S16. 1H NMR spectrum of (3-hydroxy-5-methoxy-2-oxo-2,3-dihydro-1H-indol-3-yl)acetic acid (**3c**) 18
S17. 13C NMR spectrum of (3-hydroxy-5-methoxy-2-oxo-2,3-dihydro-1H-indol-3-yl)acetic acid (**3c**) 19
S18. 1H NMR spectrum of (3-hydroxy-5-methoxy-4-nitro-2-oxo-2,3-dihydro-1H-indol-3-yl)acetic acid (**3d**) 20
S19. 1H NMR spectrum of (3-hydroxy-5-methoxy-7-nitro-2-oxo-2,3-dihydro-1H-indol-3-yl)acetic acid (**3e**) 21
S20. 13C NMR spectrum of (3-hydroxy-5-methoxy-7-nitro-2-oxo-2,3-dihydro-1H-indol-3-yl)acetic acid (**3e**) 22
S21. 1H NMR spectrum of (3-hydroxy-5,7-dinitro-2-oxo-2,3-dihydro-1H-indol-3-yl)acetic acid (**3f**) 23
S22. 1H NMR spectrum of (3-hydroxy-2-oxo-2,3-dihydro-1H-indol-3-yl)acetic acid (**3g**) 24
S23. 13C NMR spectrum of (3-hydroxy-2-oxo-2,3-dihydro-1H-indol-3-yl)acetic acid (**3g**) 25
S24. 1H NMR spectrum of (3-hydroxy-2-oxo-2,3-dihydro-1H-indol-3-yl)malonic acid (**X**) 26
S25. 13C NMR spectrum of (3-hydroxy-2-oxo-2,3-dihydro-1H-indol-3-yl)malonic acid (**X**) 27
S26. 1H NMR spectrum of (5-bromo-3-hydroxy-2-oxo-2,3-dihydro-1H-indol-3-yl)acetic acid (**3h**) 28
S27. 13C NMR spectrum of (5-bromo-3-hydroxy-2-oxo-2,3-dihydro-1H-indol-3-yl)acetic acid (**3h**) 29
S28. 1H NMR spectrum of (1-benzyl-3-hydroxy-5-methoxy-2-oxo-2,3-dihydro-1H-indol-3-yl)acetic acid (**3i**) 30
S29. 13C NMR spectrum of (1-benzyl-3-hydroxy-5-methoxy-2-oxo-2,3-dihydro-1H-indol-3-yl)acetic acid (**3i**) 31
HPLC data of obtained compounds 32
S30 (3-hydroxy-5-nitro-2-oxo-2,3-dihydro-1H-indol-3-yl)acetonitrile (2a) 32
S31 (3-hydroxy-7-nitro-2-oxo-2,3-dihydro-1H-indol-3-yl)acetonitrile (2b) 33
S32 (3-hydroxy-5-methoxy-7-nitro-2-oxo-2,3-dihydro-1H-indol-3-yl)acetonitrile (**2e**) 34
S33 (3-hydroxy-5,7-dinitro-2-oxo-2,3-dihydro-1H-indol-3-yl)acetonitrile (**2f**) 35
S34 (5-bromo-3-hydroxy-2-oxo-2,3-dihydro-1H-indol-3-yl)acetonitrile (**2h**) 36

S1. 1H NMR spectrum of (3-hydroxy-5-nitro-2-oxo-2,3-dihydro-1H-indol-3-yl)acetonitrile (**2a**)

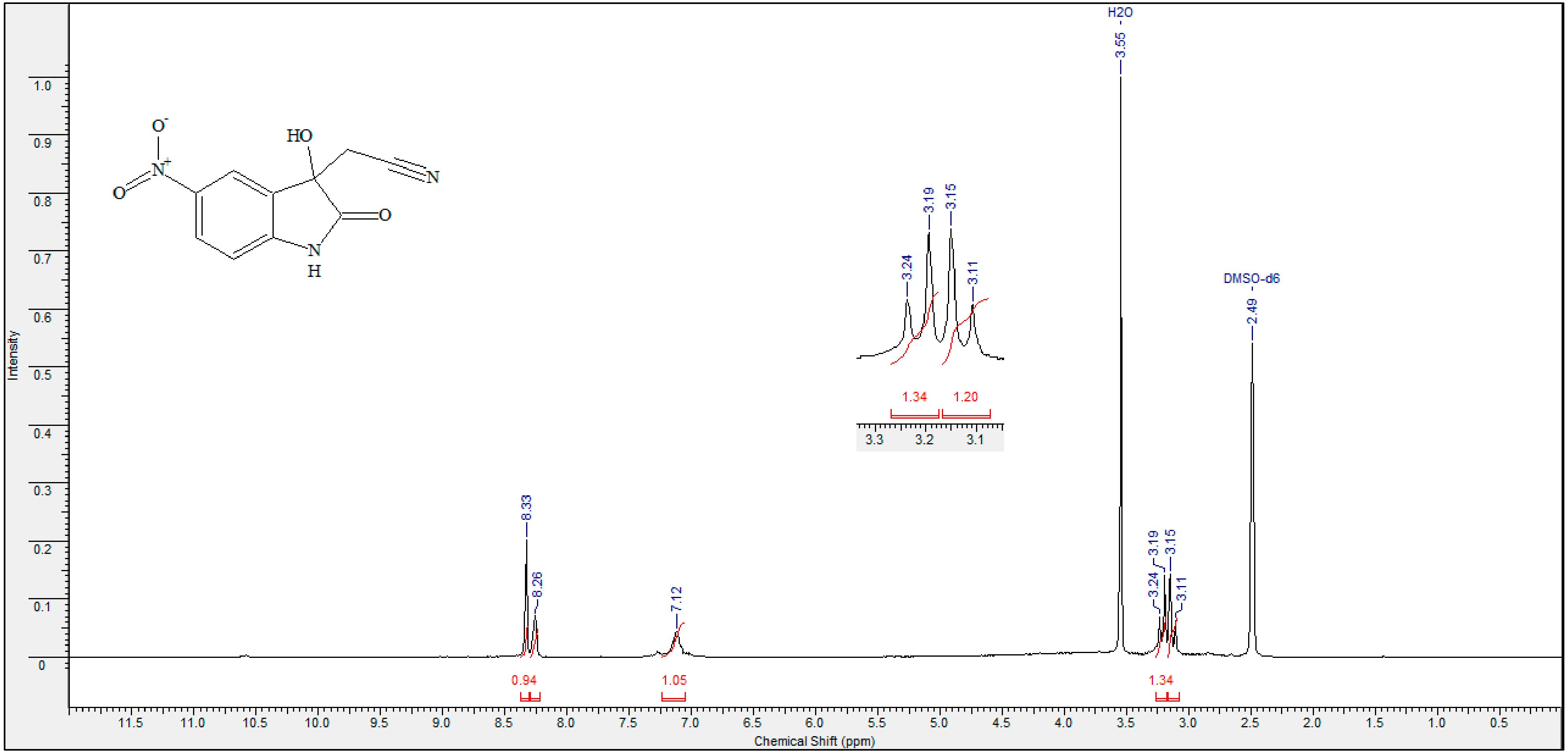

S2. 1H NMR spectrum of (3-hydroxy-7-nitro-2-oxo-2,3-dihydro-1H-indol-3-yl)acetonitrile (2b)

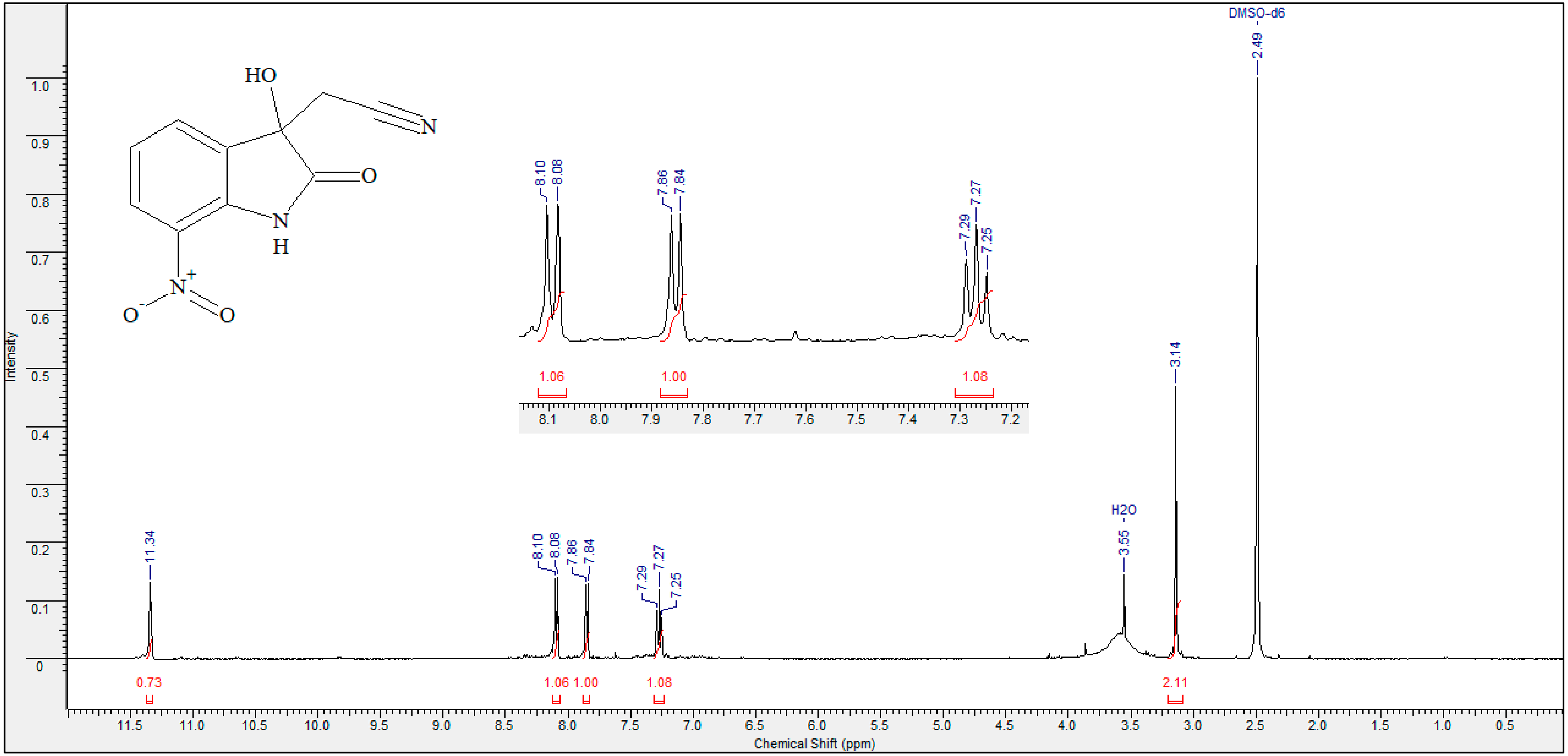

S3. 1H NMR spectrum of (3-hydroxy-5-methoxy-2-oxo-2,3-dihydro-1H-indol-3-yl)acetonitrile (**2c**)

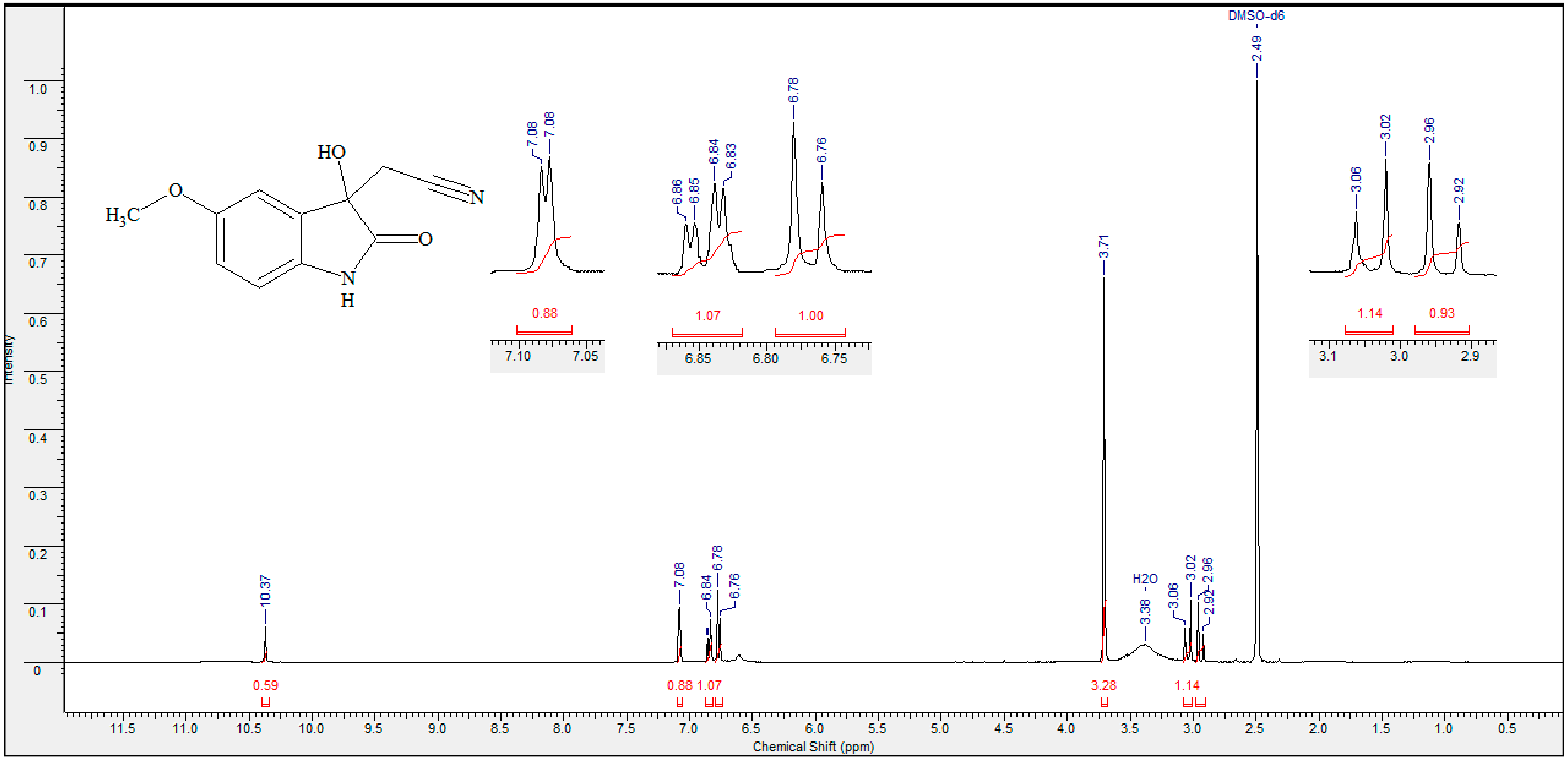

S4. 13C NMR spectrum of (3-hydroxy-5-methoxy-2-oxo-2,3-dihydro-1H-indol-3-yl)acetonitrile (2c)

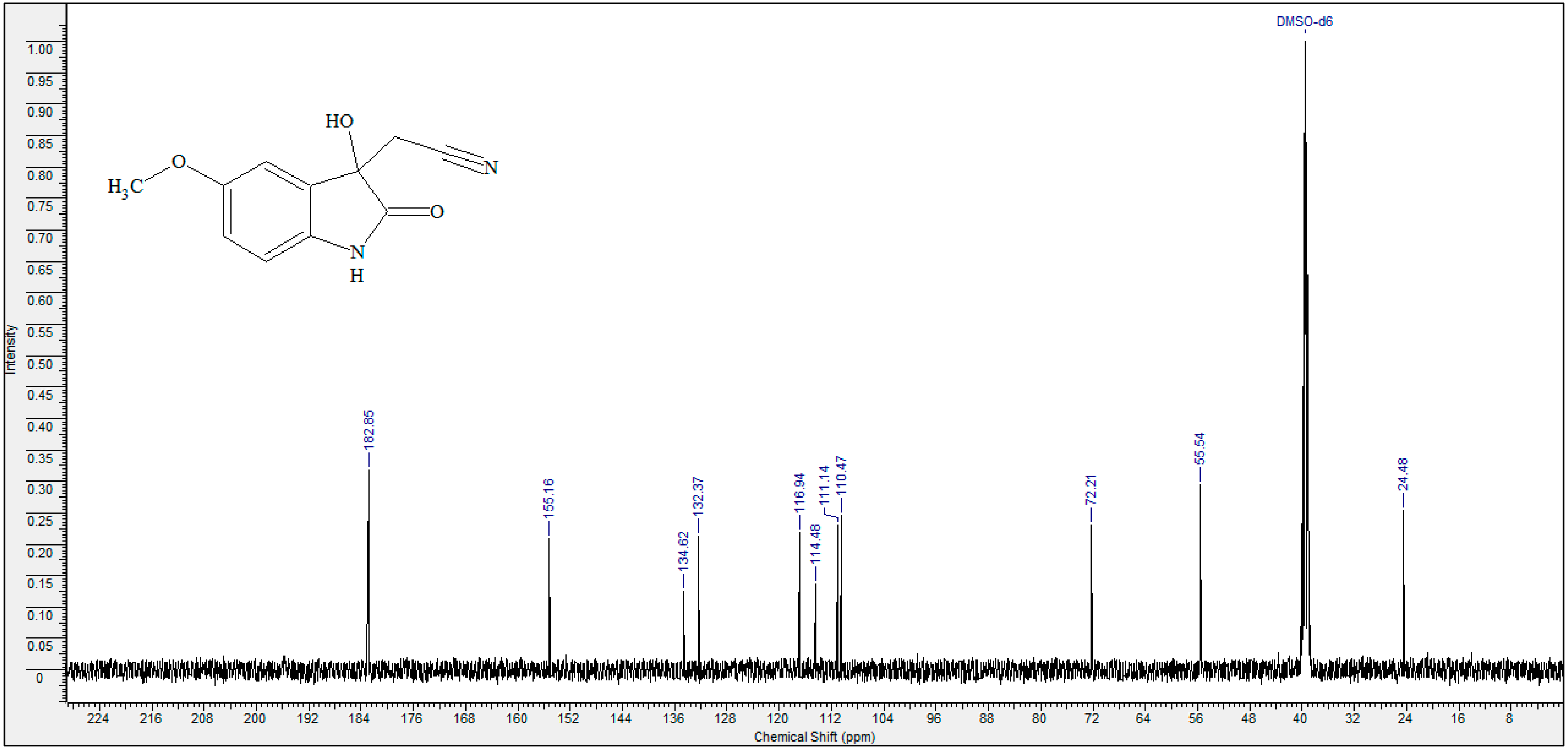

S5. 1H NMR spectrum of (3-hydroxy-5-methoxy-4-nitro-2-oxo-2,3-dihydro-1H-indol-3-yl)acetonitrile (**2d**)

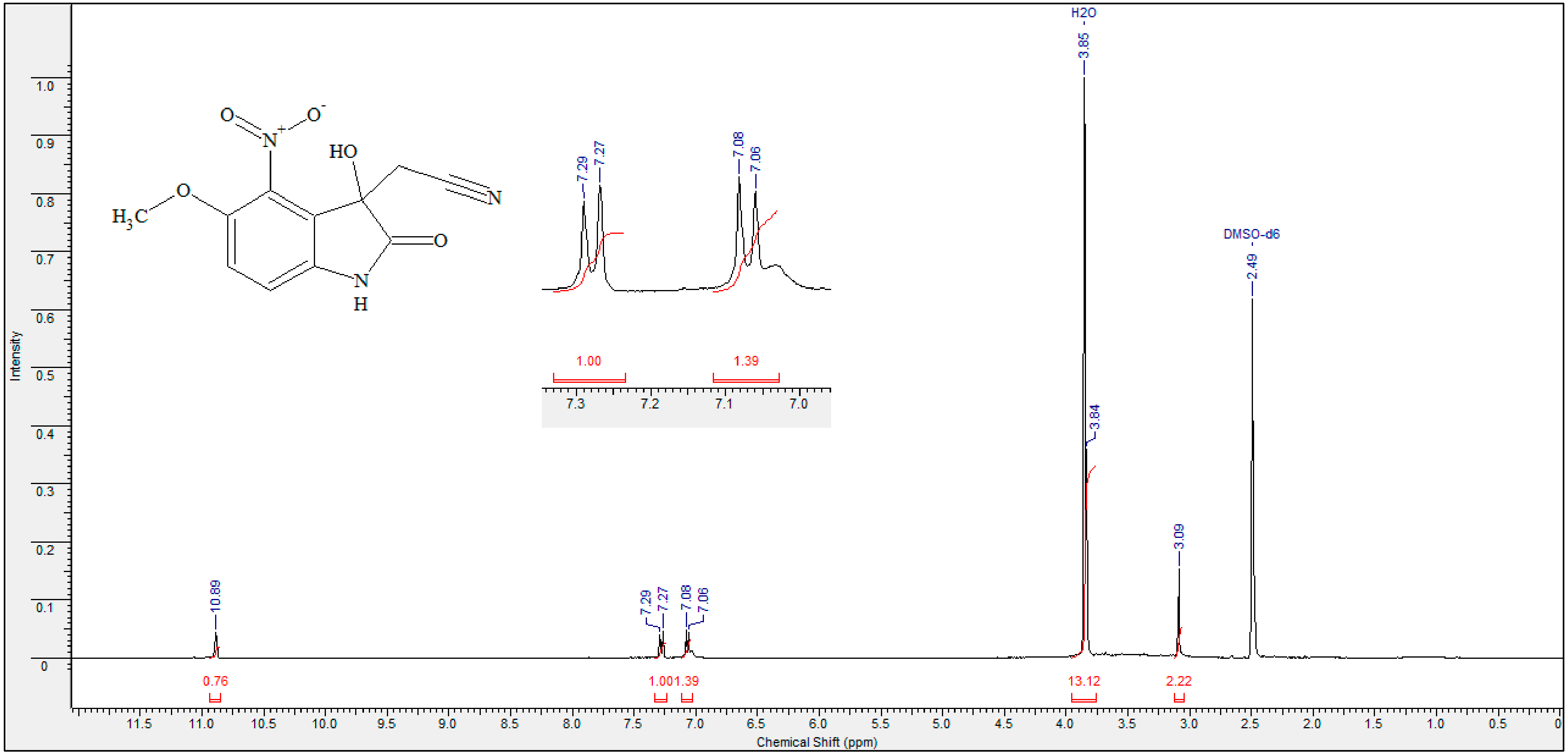

S6. 1H NMR spectrum of (3-hydroxy-5-methoxy-7-nitro-2-oxo-2,3-dihydro-1H-indol-3-yl)acetonitrile (**2e**)

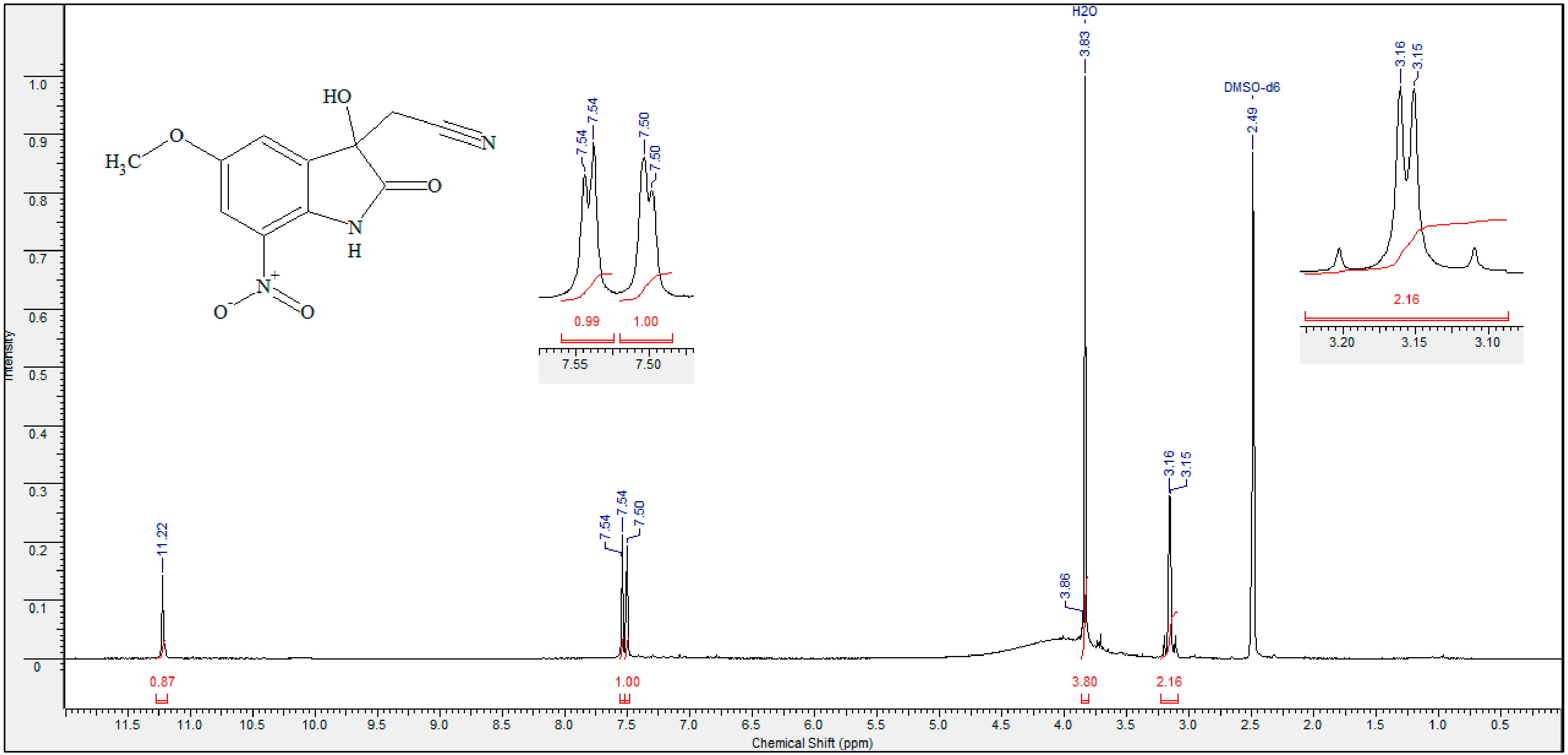

S7. 13C NMR spectrum of (3-hydroxy-5-methoxy-7-nitro-2-oxo-2,3-dihydro-1H-indol-3-yl)acetonitrile (**2e**)

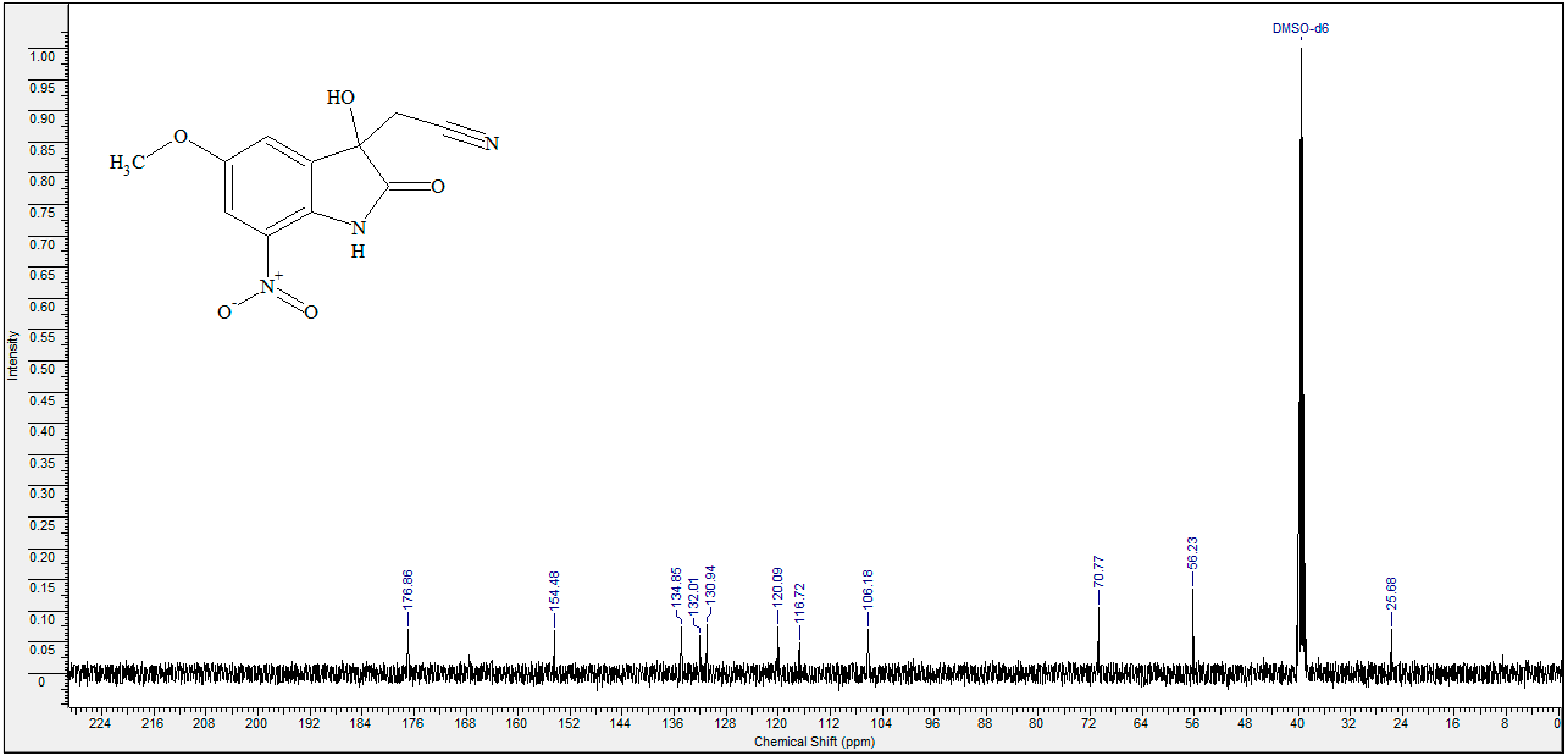

S8. 1H NMR spectrum of (3-hydroxy-5,7-dinitro-2-oxo-2,3-dihydro-1H-indol-3-yl)acetonitrile (**2f**)

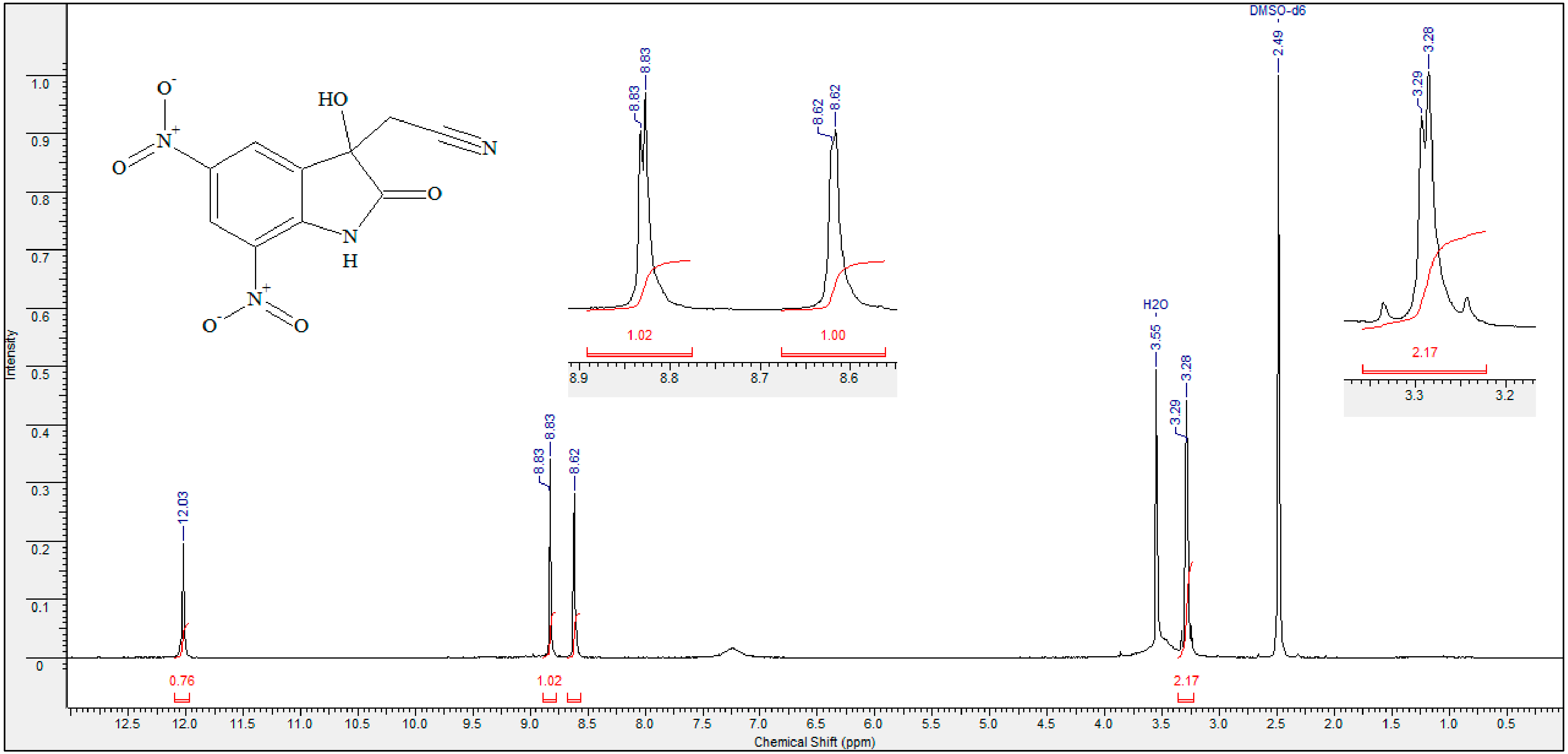

S9. 1H NMR spectrum of (3-hydroxy-2-oxo-2,3-dihydro-1H-indol-3-yl)acetonitrile (**2g**)

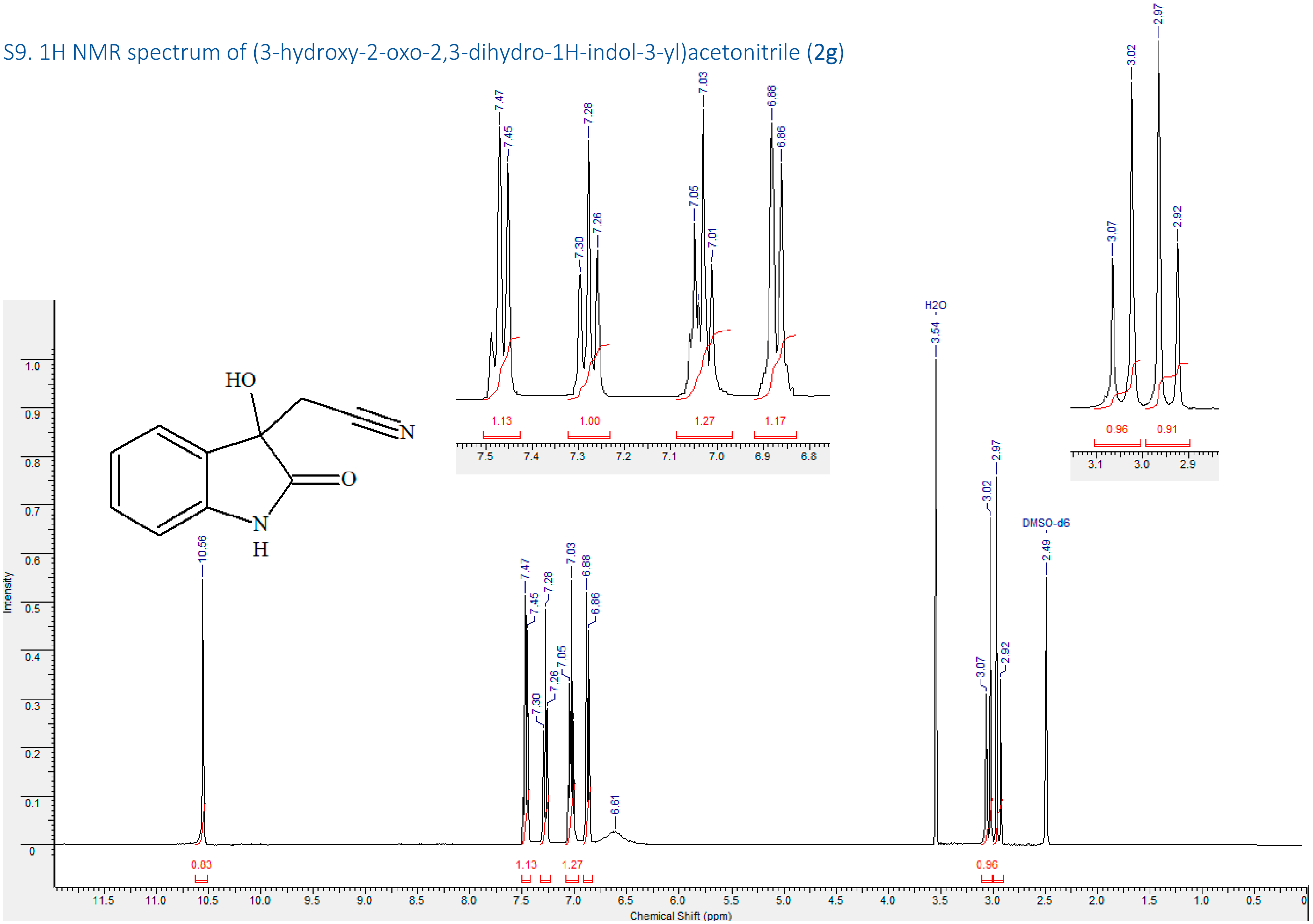

S10. 1H NMR spectrum of (5-bromo-3-hydroxy-2-oxo-2,3-dihydro-1H-indol-3-yl)acetonitrile (**2h**)

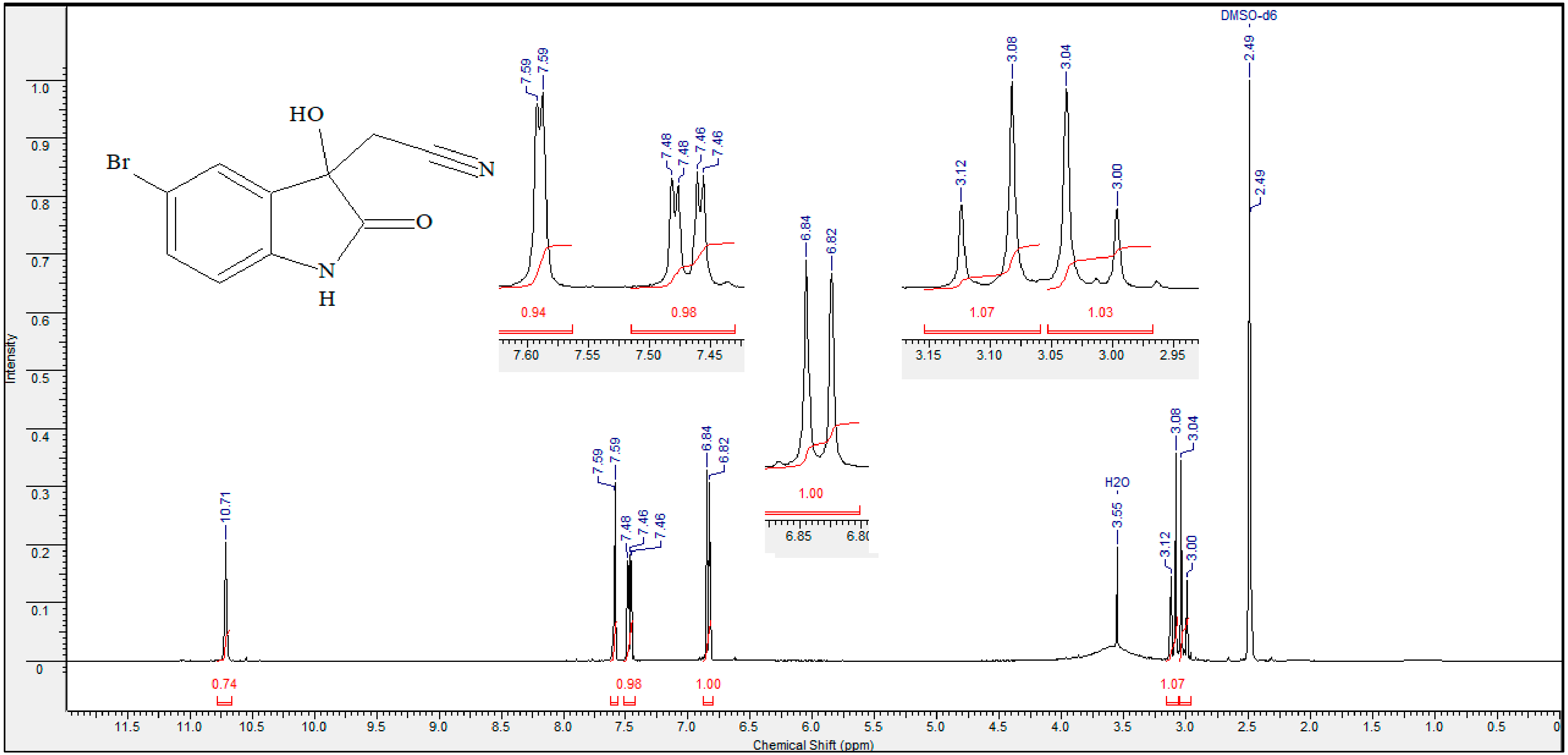

S11. 13C NMR spectrum of (5-bromo-3-hydroxy-2-oxo-2,3-dihydro-1H-indol-3-yl)acetonitrile (**2h**)

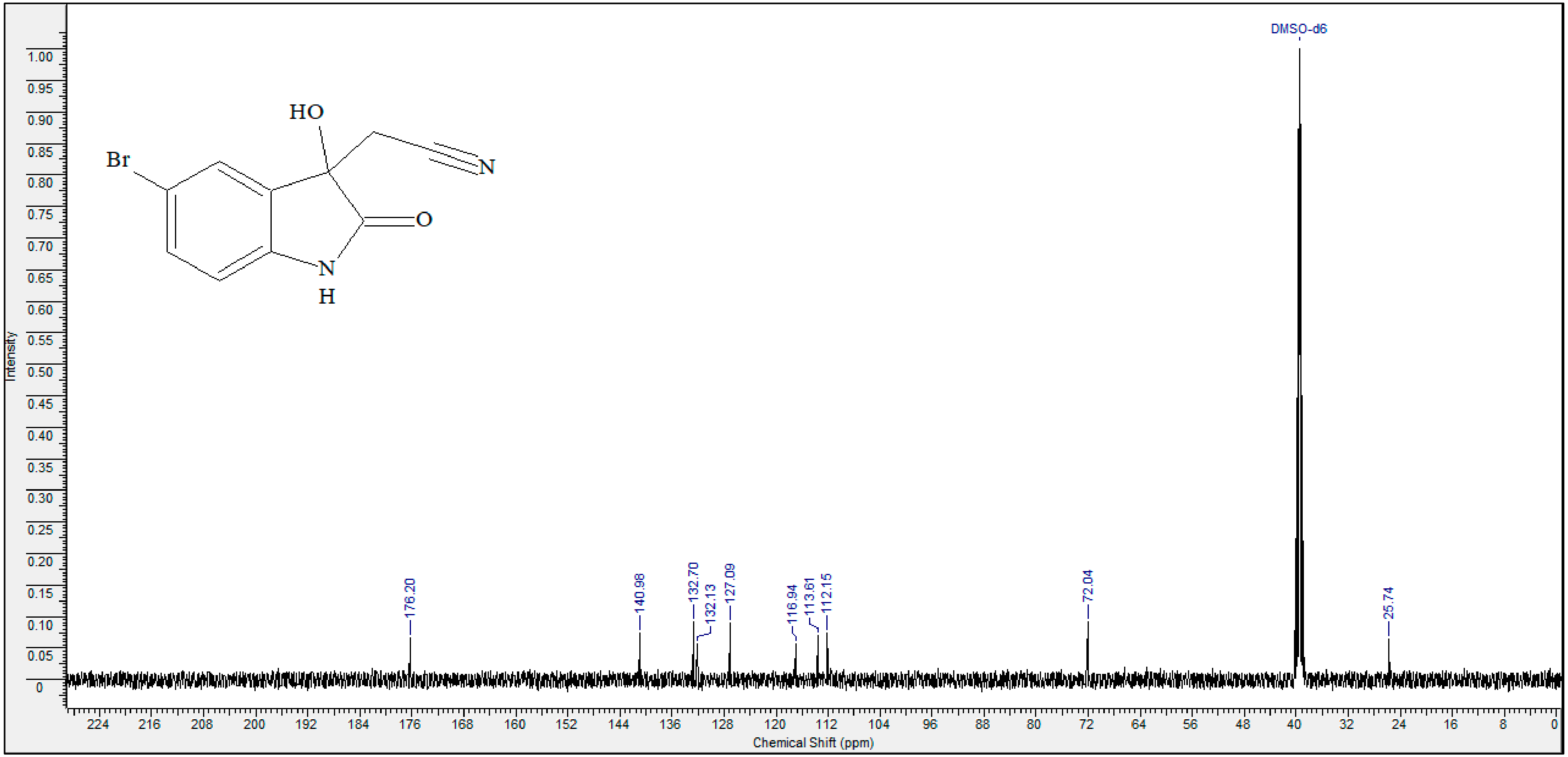

S12. 1H NMR spectrum of (1-benzyl-3-hydroxy-5-methoxy-2-oxo-2,3-dihydro-1H-indol-3-yl)acetonitrile (**2i**)

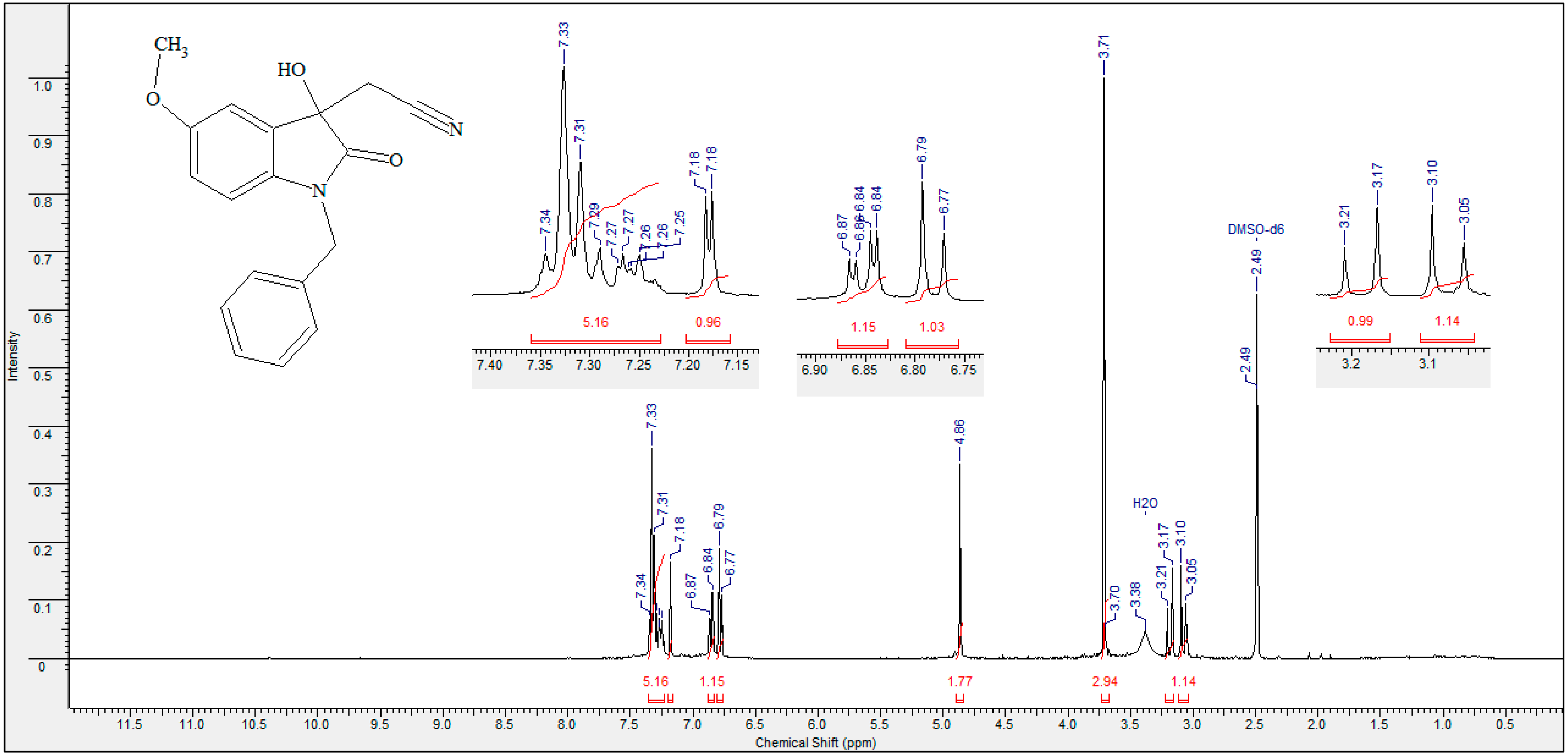

S13. 13C NMR spectrum of (1-benzyl-3-hydroxy-5-methoxy-2-oxo-2,3-dihydro-1H-indol-3-yl)acetonitrile (2i)

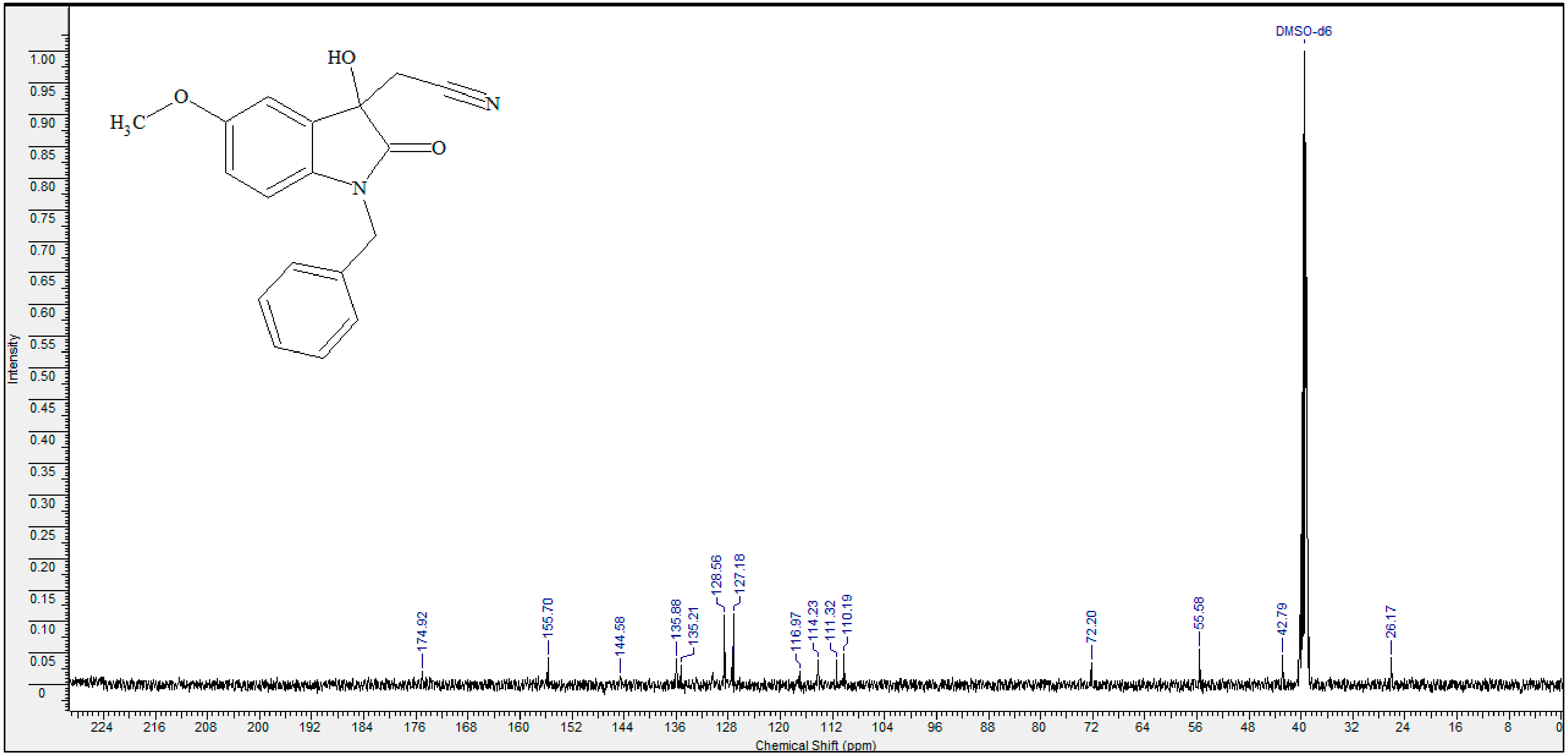

S14. 1H NMR spectrum of (3-hydroxy-5-nitro-2-oxo-2,3-dihydro-1H-indol-3-yl)acetic acid (**3a**)

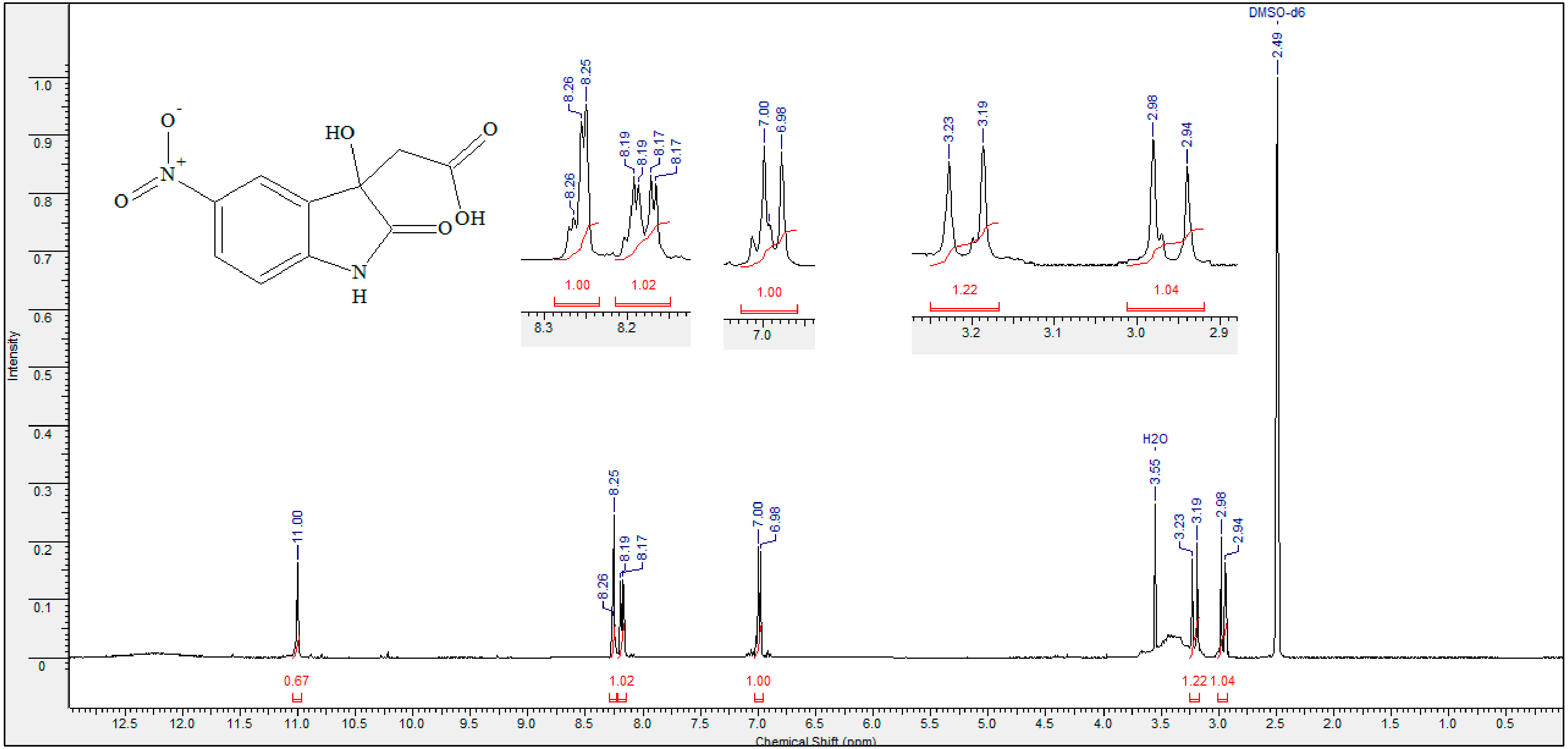

S15. 1H NMR spectrum of (3-hydroxy-7-nitro-2-oxo-2,3-dihydro-1H-indol-3-yl)acetic acid (**3b**)

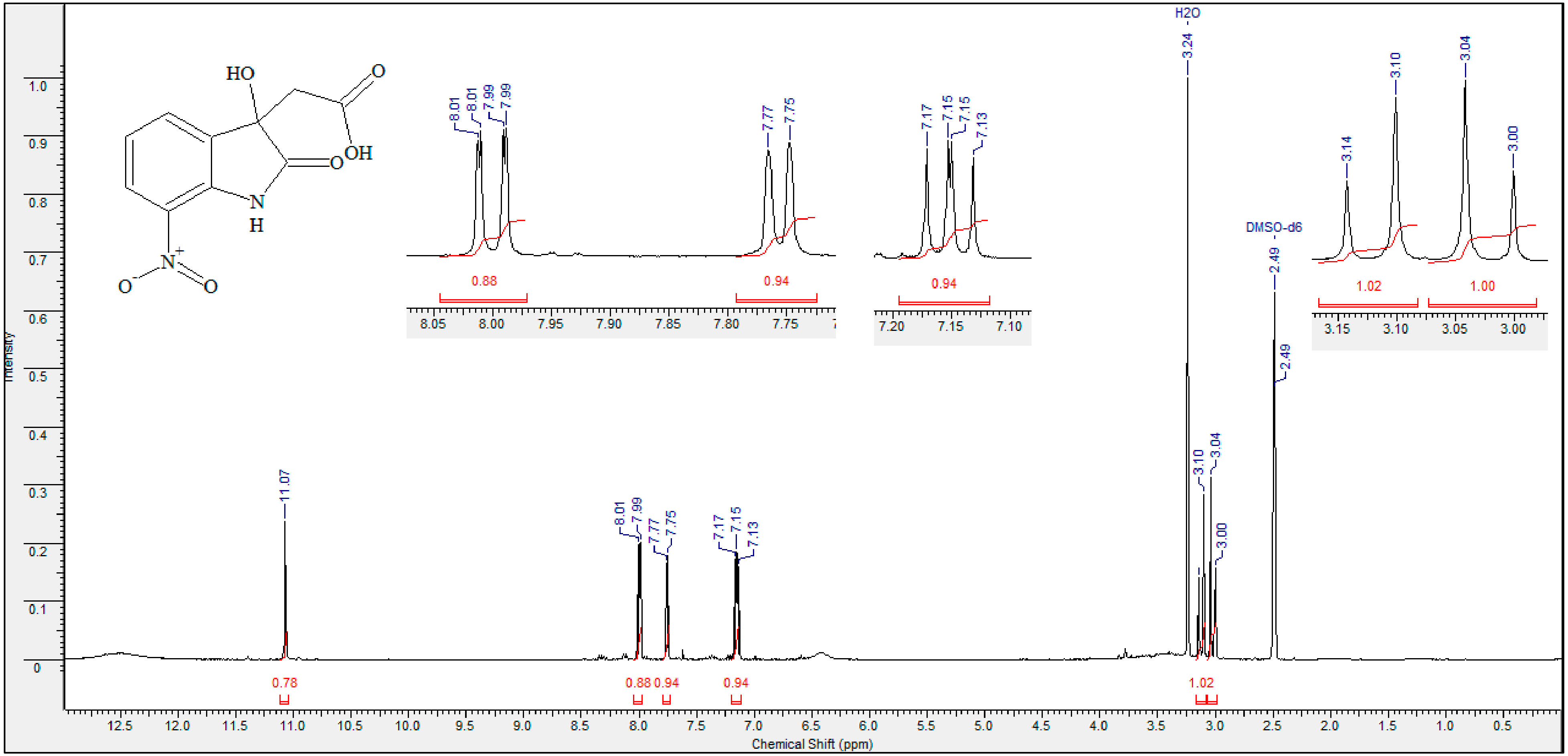

S16. 1H NMR spectrum of (3-hydroxy-5-methoxy-2-oxo-2,3-dihydro-1H-indol-3-yl)acetic acid (**3c**)

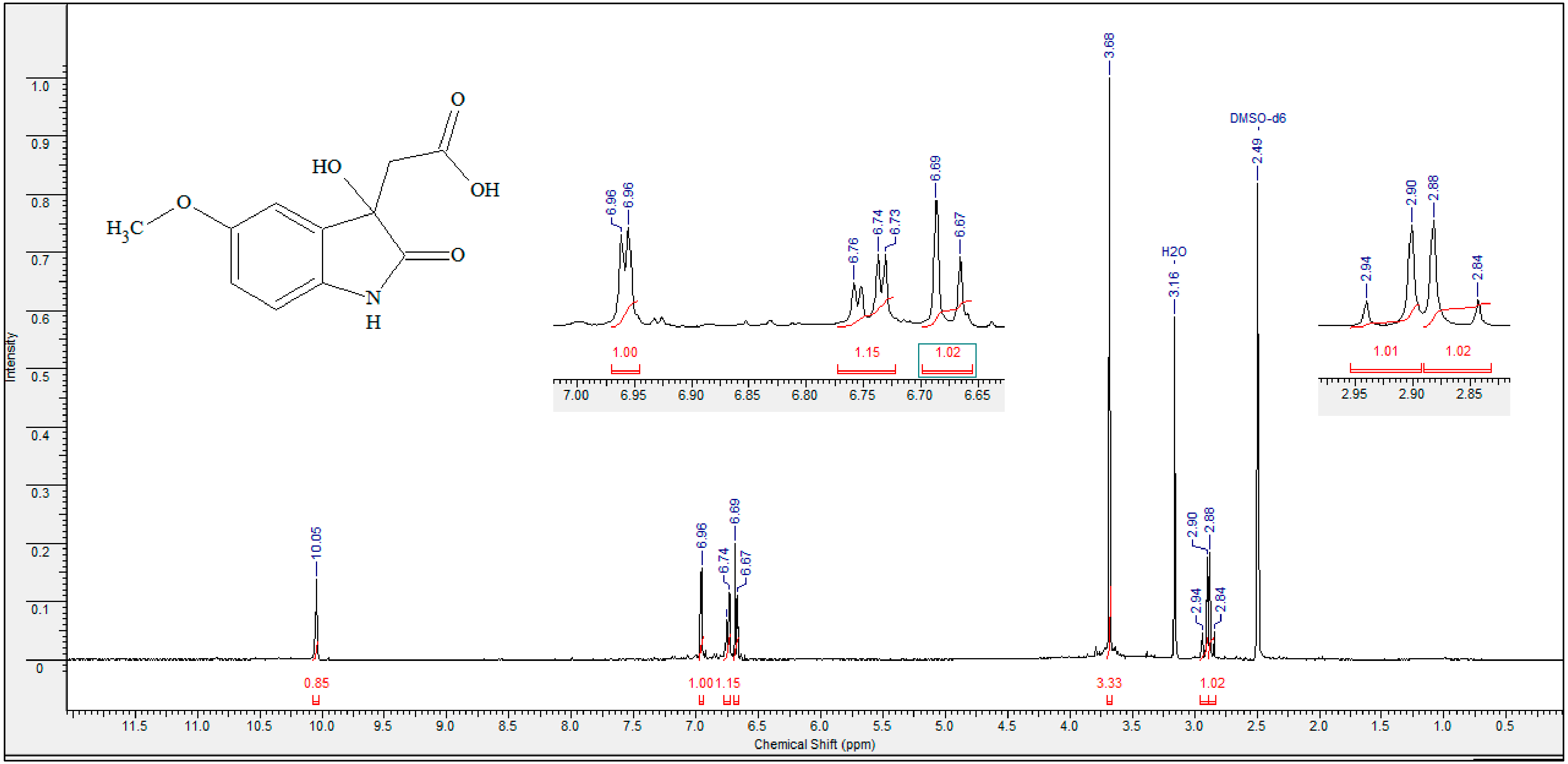

S17. 13C NMR spectrum of (3-hydroxy-5-methoxy-2-oxo-2,3-dihydro-1H-indol-3-yl)acetic acid (3c)

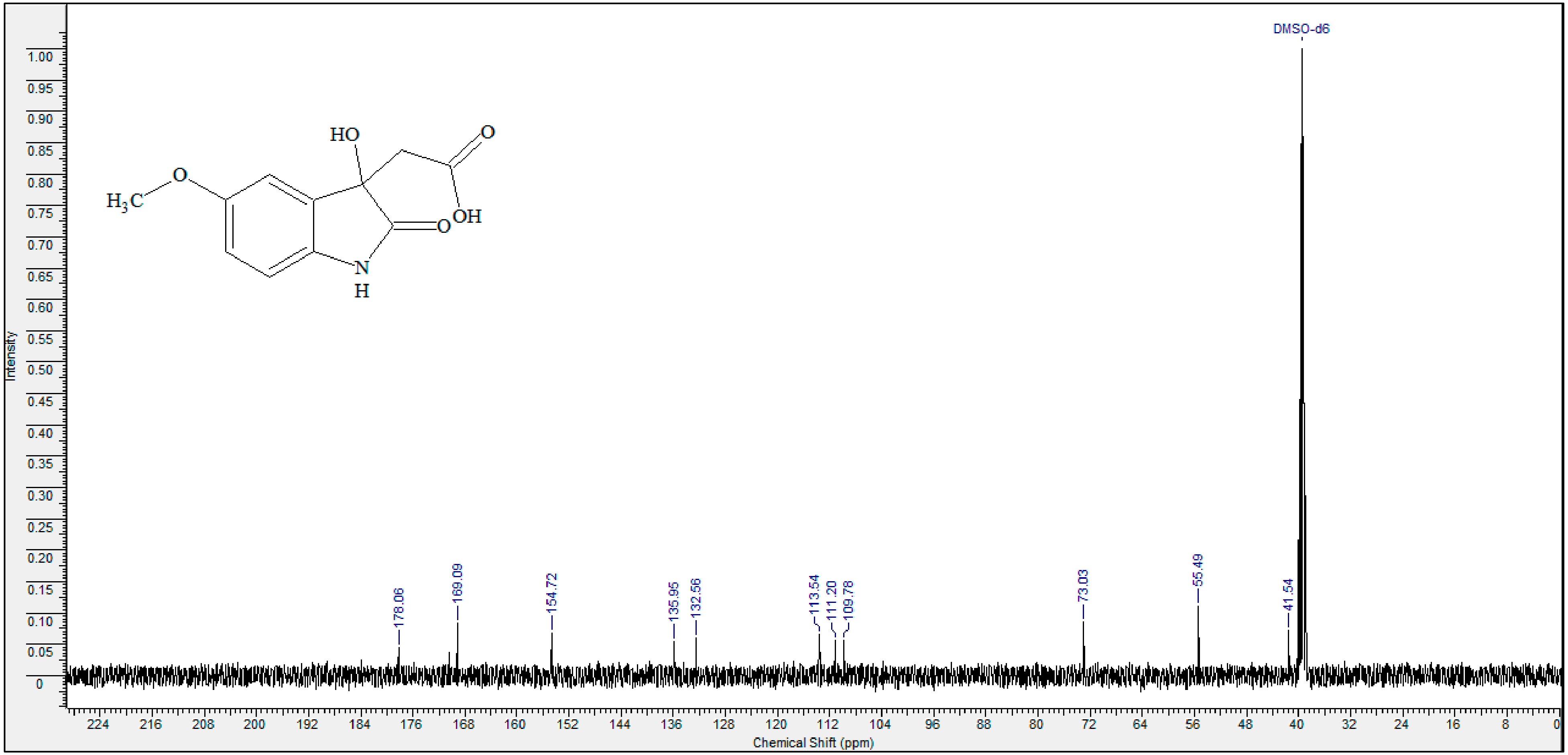

S18. 1H NMR spectrum of (3-hydroxy-5-methoxy-4-nitro-2-oxo-2,3-dihydro-1H-indol-3-yl)acetic acid (**3d**)

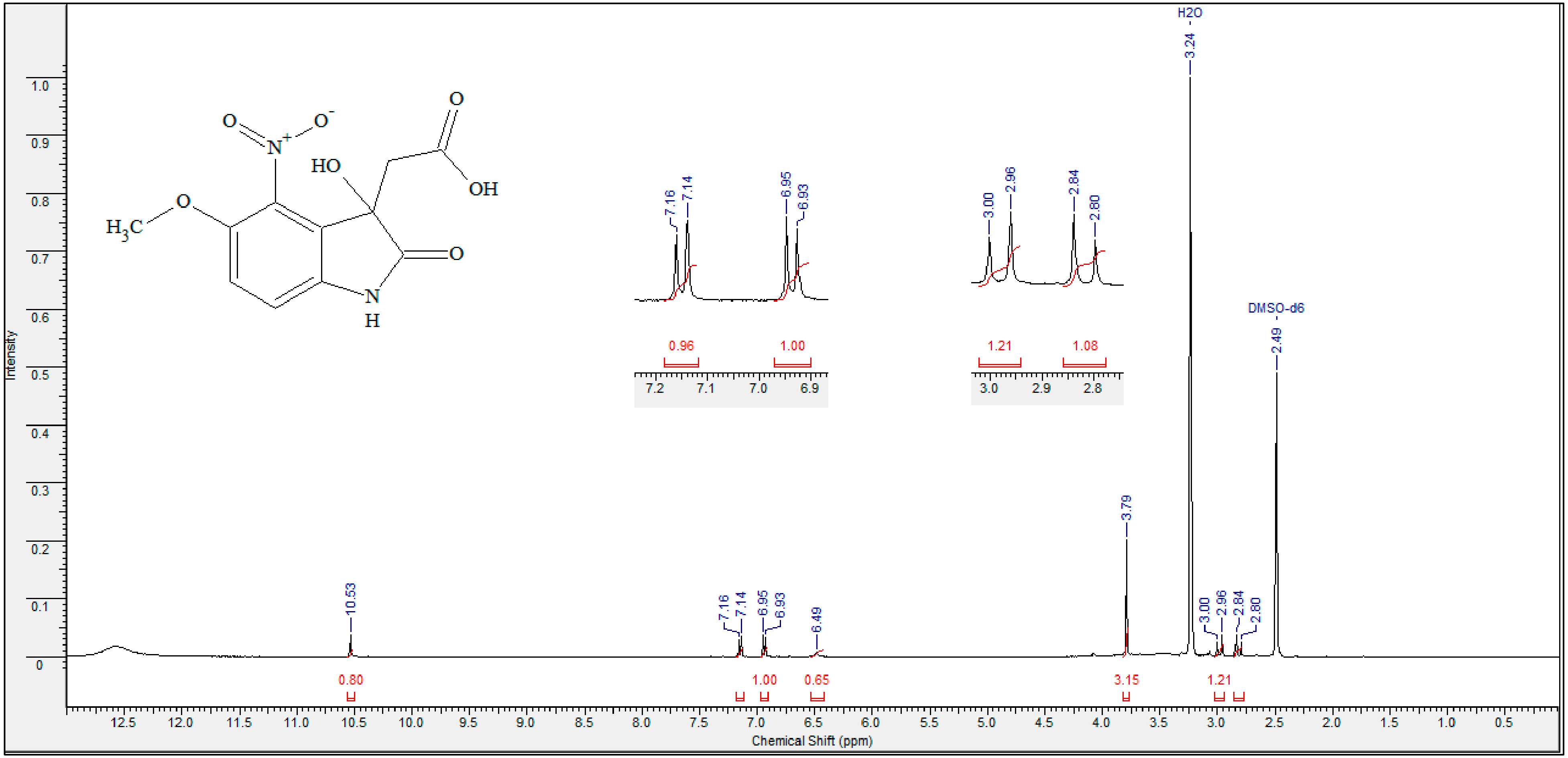

S19. 1H NMR spectrum of (3-hydroxy-5-methoxy-7-nitro-2-oxo-2,3-dihydro-1H-indol-3-yl)acetic acid (**3e**)

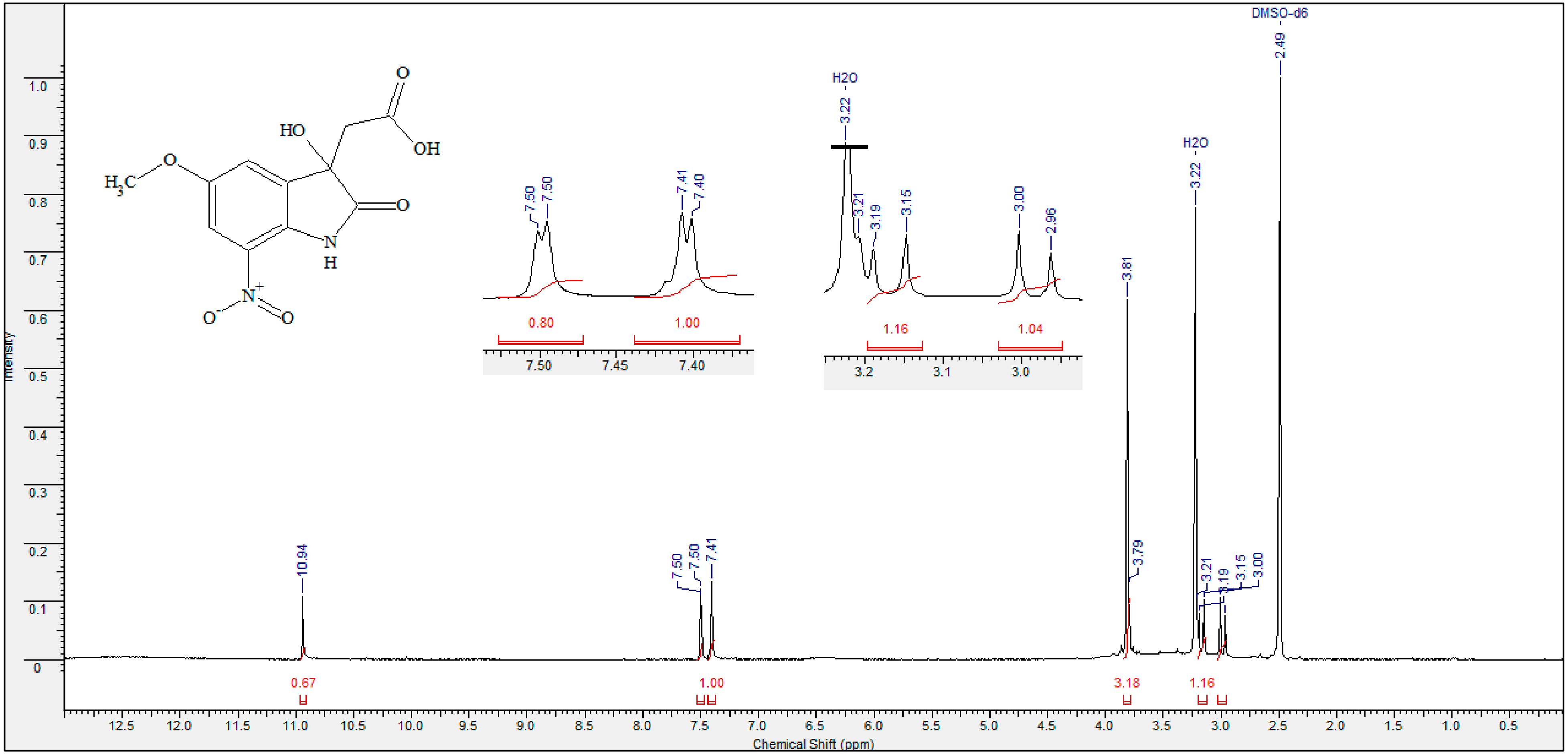

S20. 13C NMR spectrum of (3-hydroxy-5-methoxy-7-nitro-2-oxo-2,3-dihydro-1H-indol-3-yl)acetic acid (**3e**)

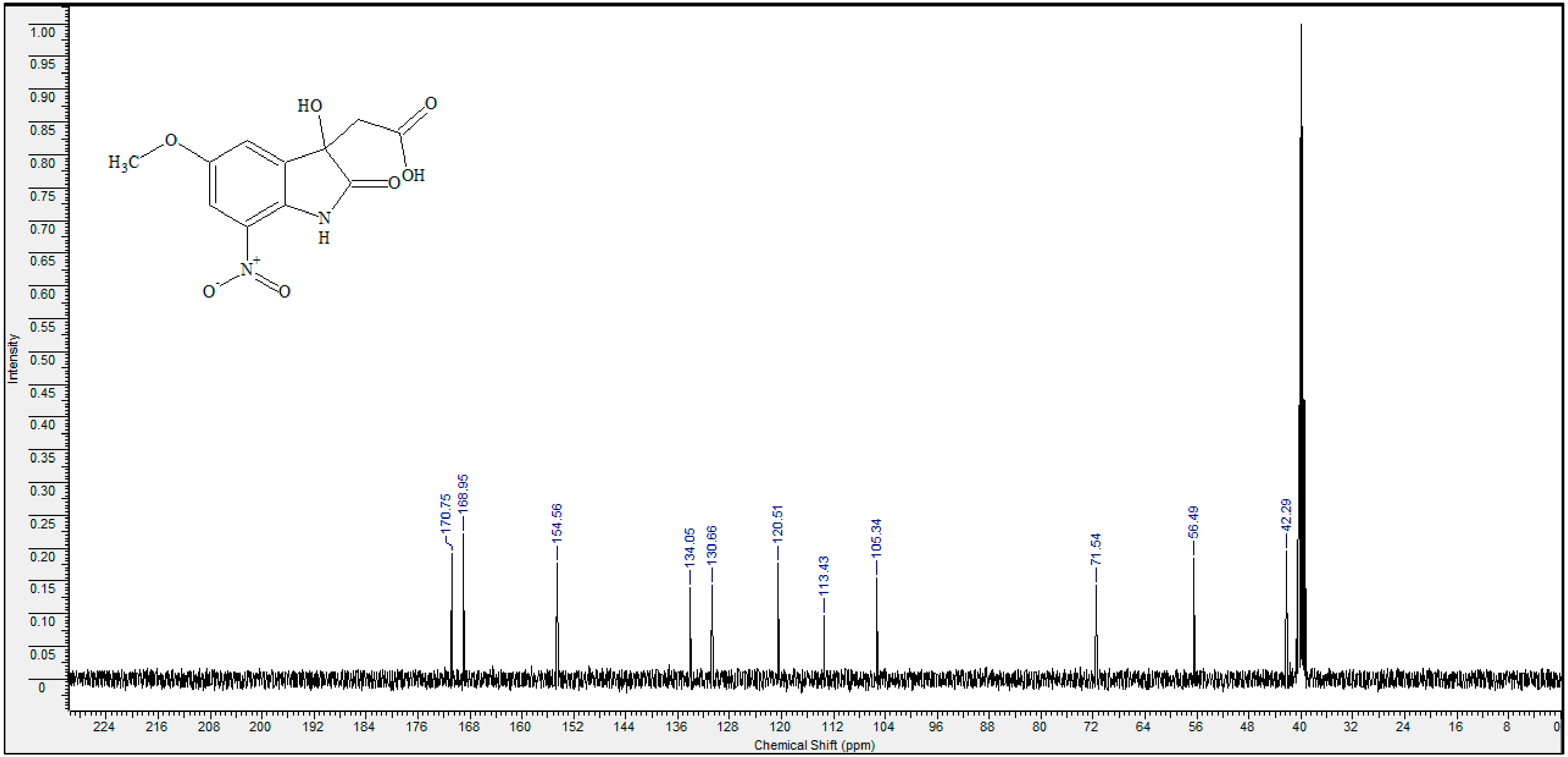

S21. 1H NMR spectrum of (3-hydroxy-5,7-dinitro-2-oxo-2,3-dihydro-1H-indol-3-yl)acetic acid (**3f**)

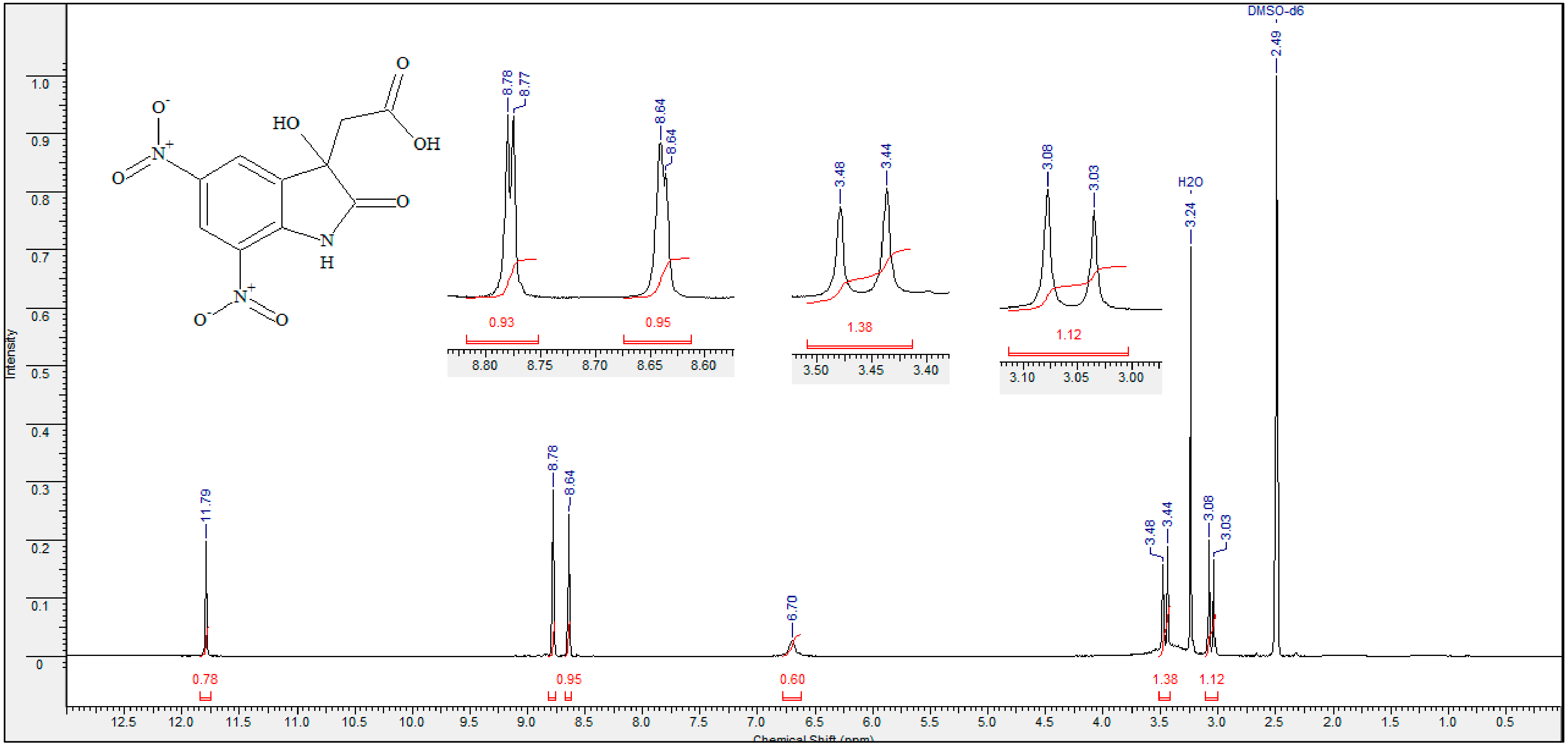

S22. 1H NMR spectrum of (3-hydroxy-2-oxo-2,3-dihydro-1H-indol-3-yl)acetic acid (**3g**)

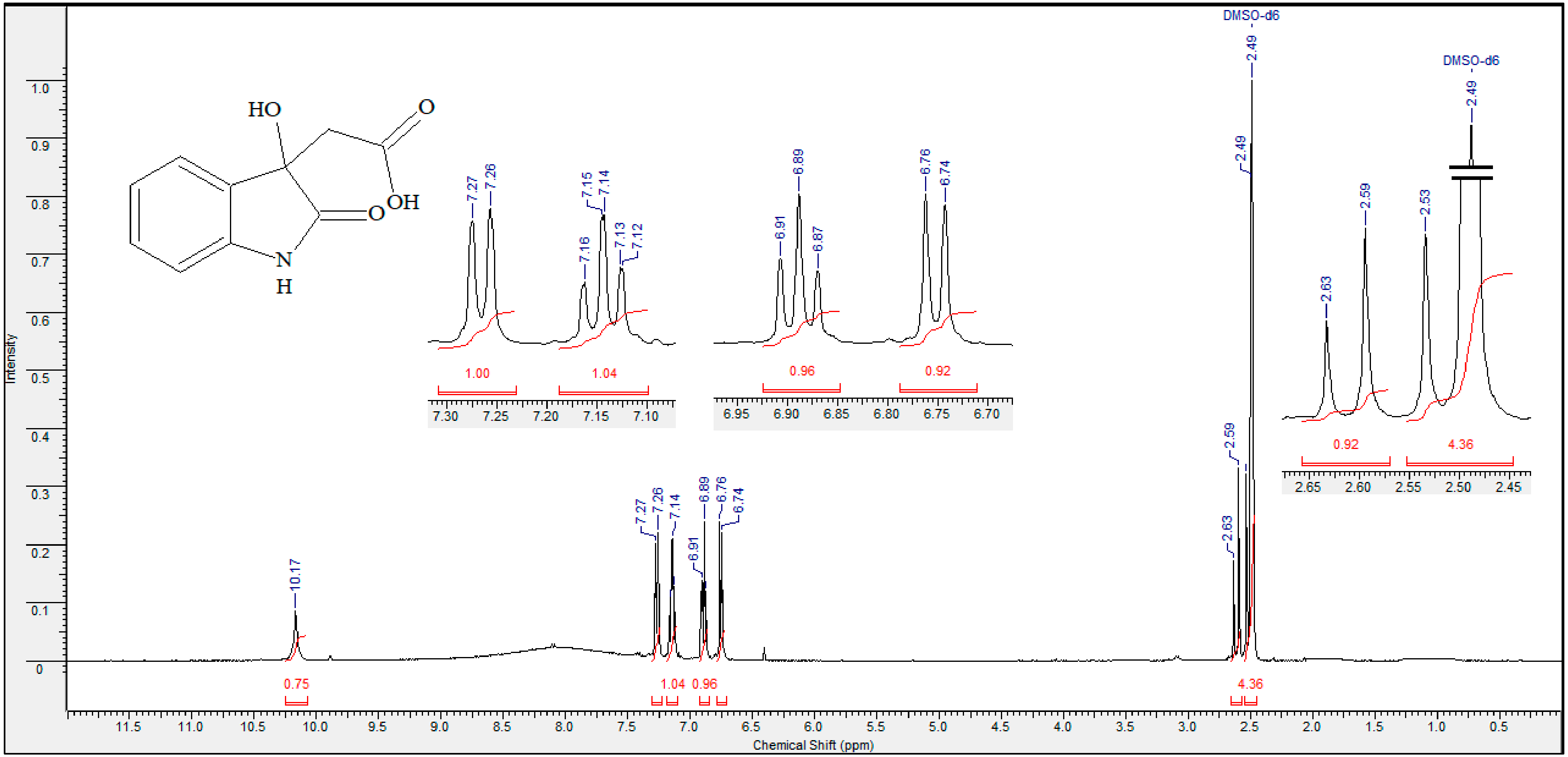

S23. 13C NMR spectrum of (3-hydroxy-2-oxo-2,3-dihydro-1H-indol-3-yl)acetic acid (**3g**)

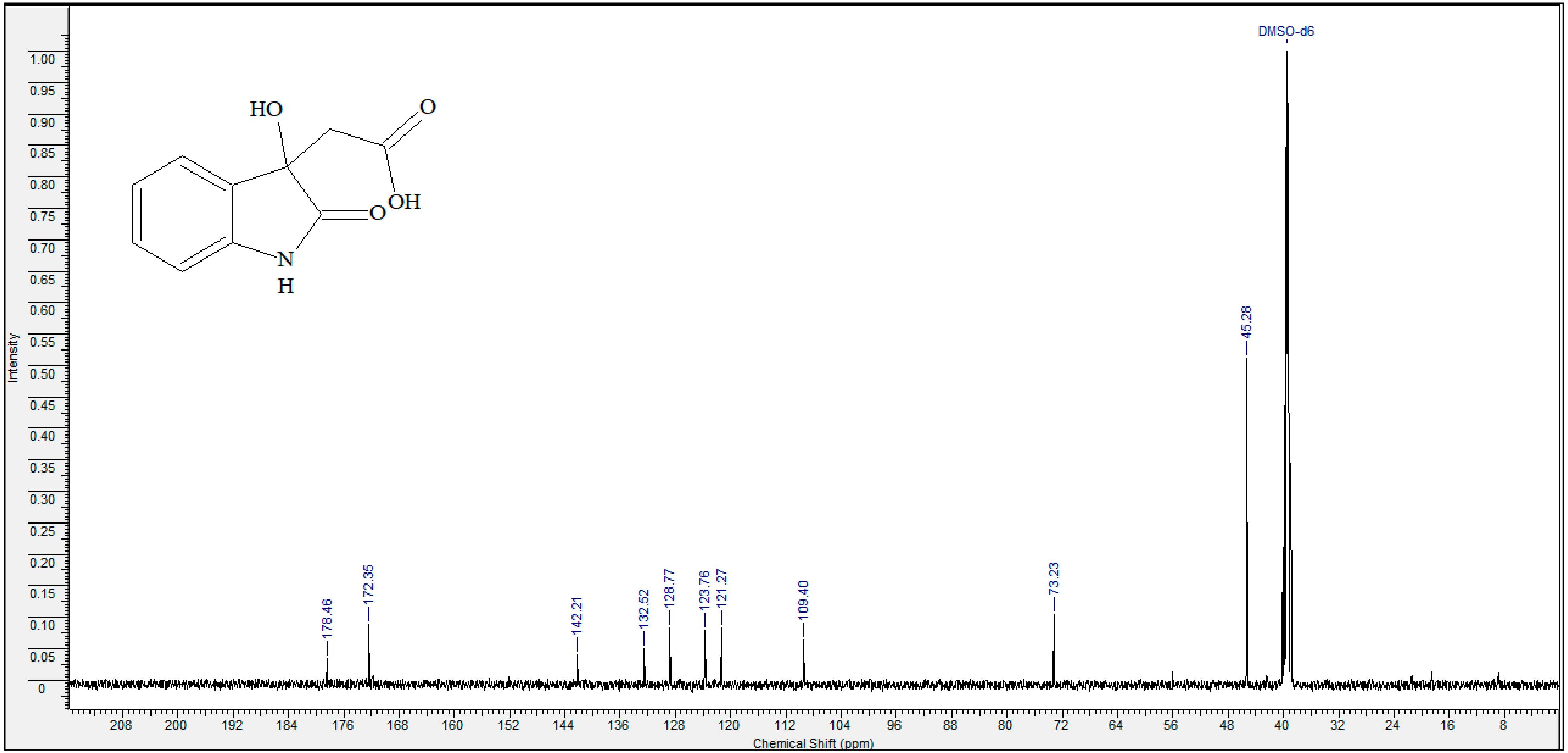

S24. 1H NMR spectrum of (3-hydroxy-2-oxo-2,3-dihydro-1H-indol-3-yl)malonic acid (X)

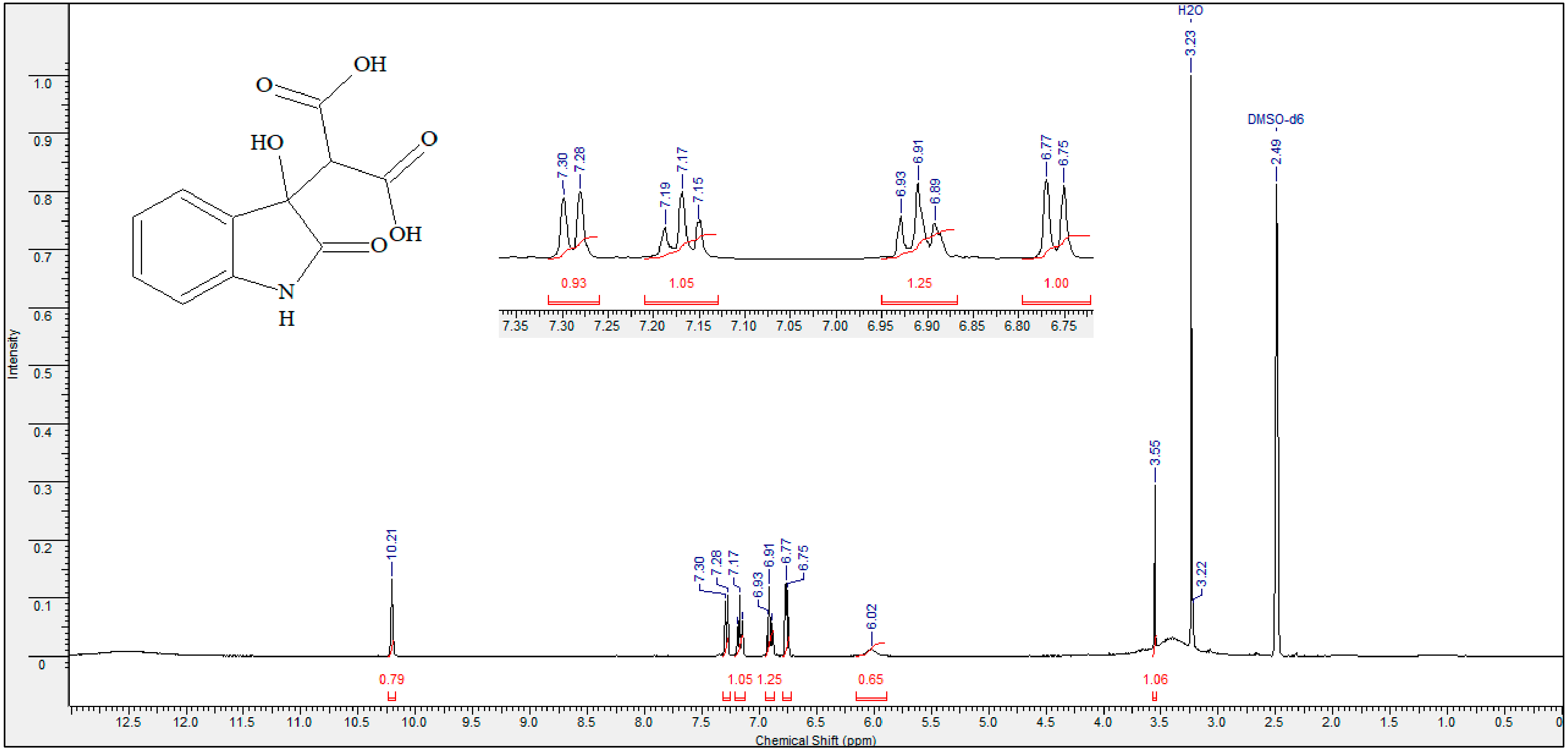

S25. 13C NMR spectrum of (3-hydroxy-2-oxo-2,3-dihydro-1H-indol-3-yl)malonic acid (X)

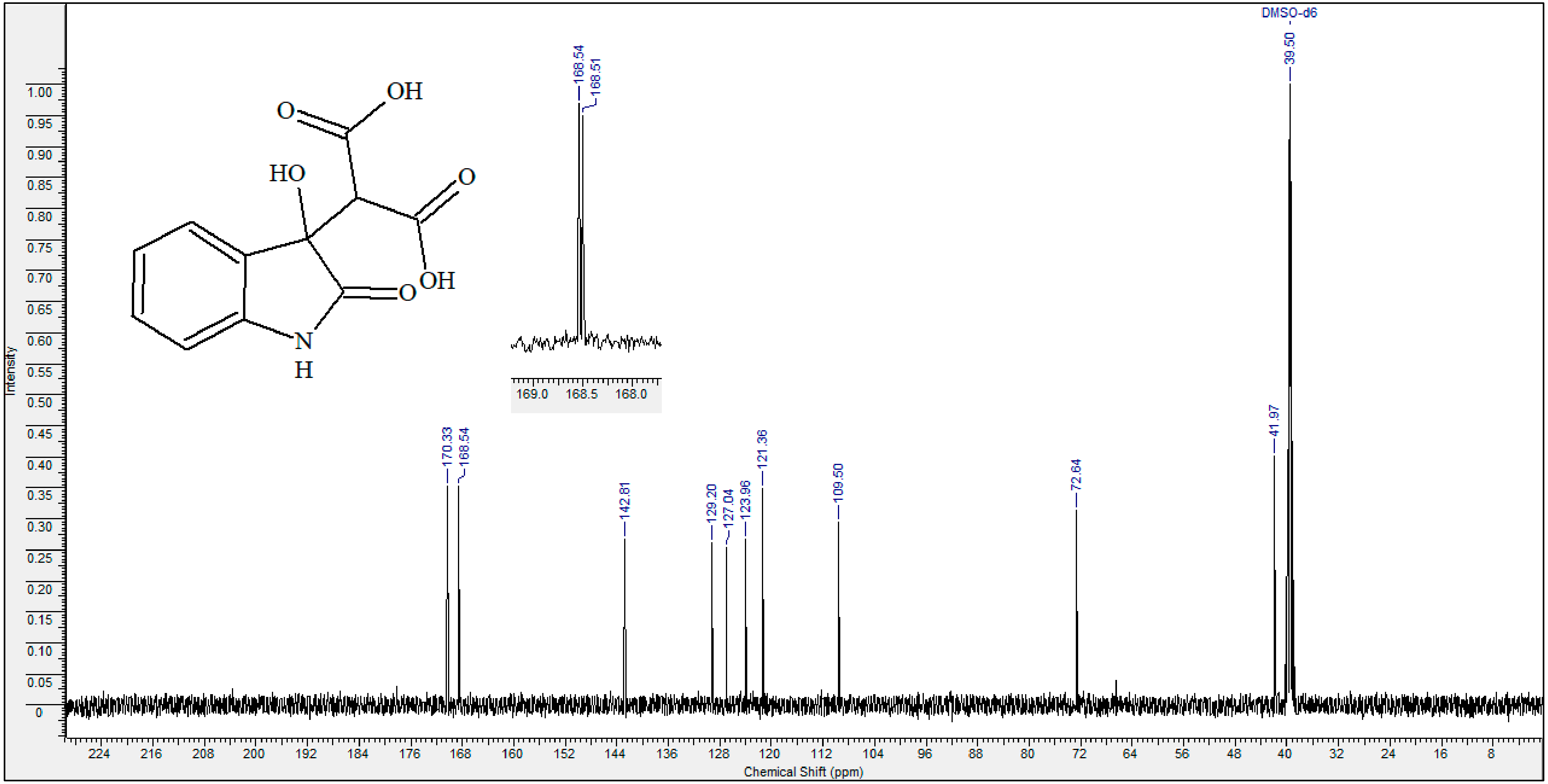

S26. 1H NMR spectrum of (5-bromo-3-hydroxy-2-oxo-2,3-dihydro-1H-indol-3-yl)acetic acid (**3h**)

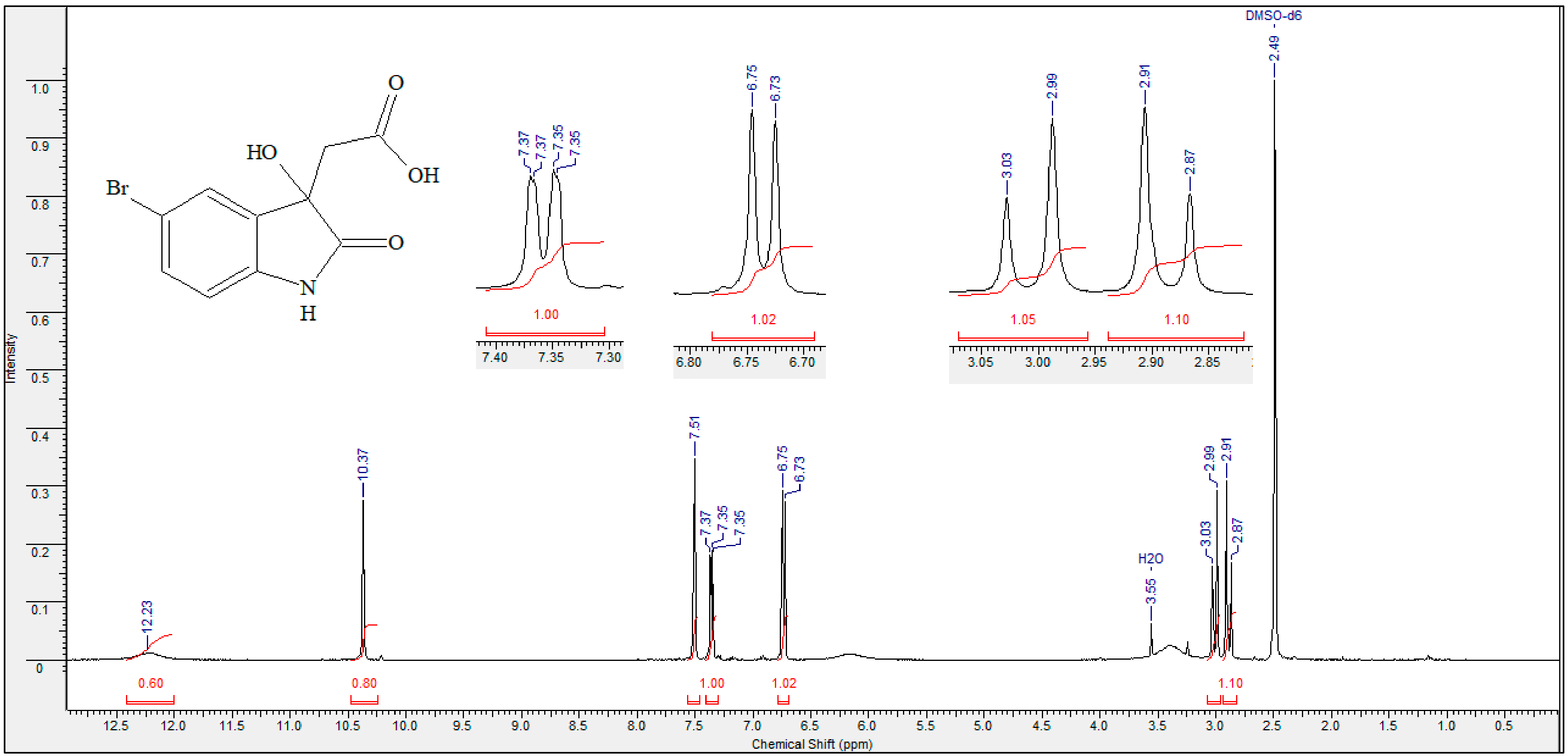

S27. 13C NMR spectrum of (5-bromo-3-hydroxy-2-oxo-2,3-dihydro-1H-indol-3-yl)acetic acid (3h)

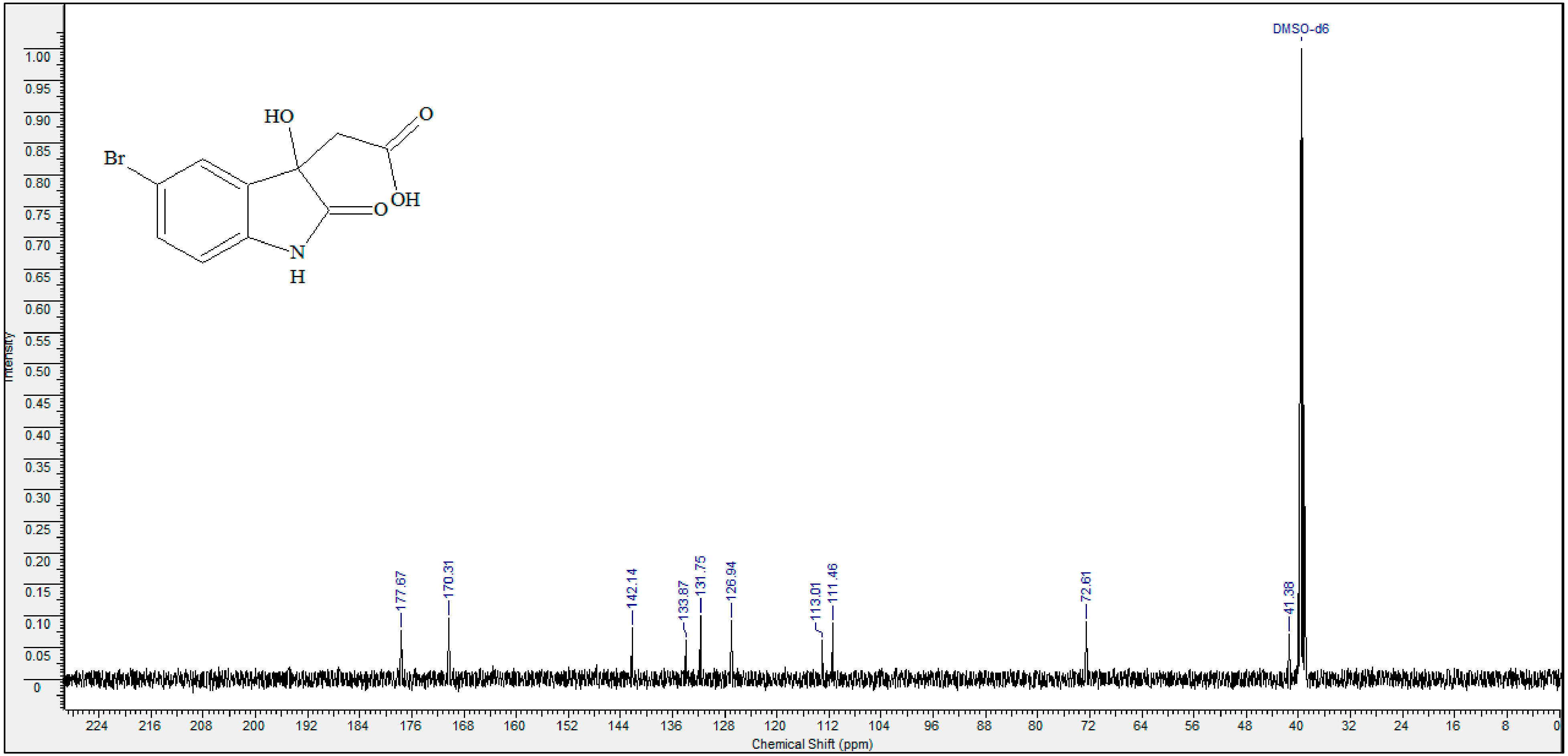

S28. 1H NMR spectrum of (1-benzyl-3-hydroxy-5-methoxy-2-oxo-2,3-dihydro-1H-indol-3-yl)acetic acid (**3i**)

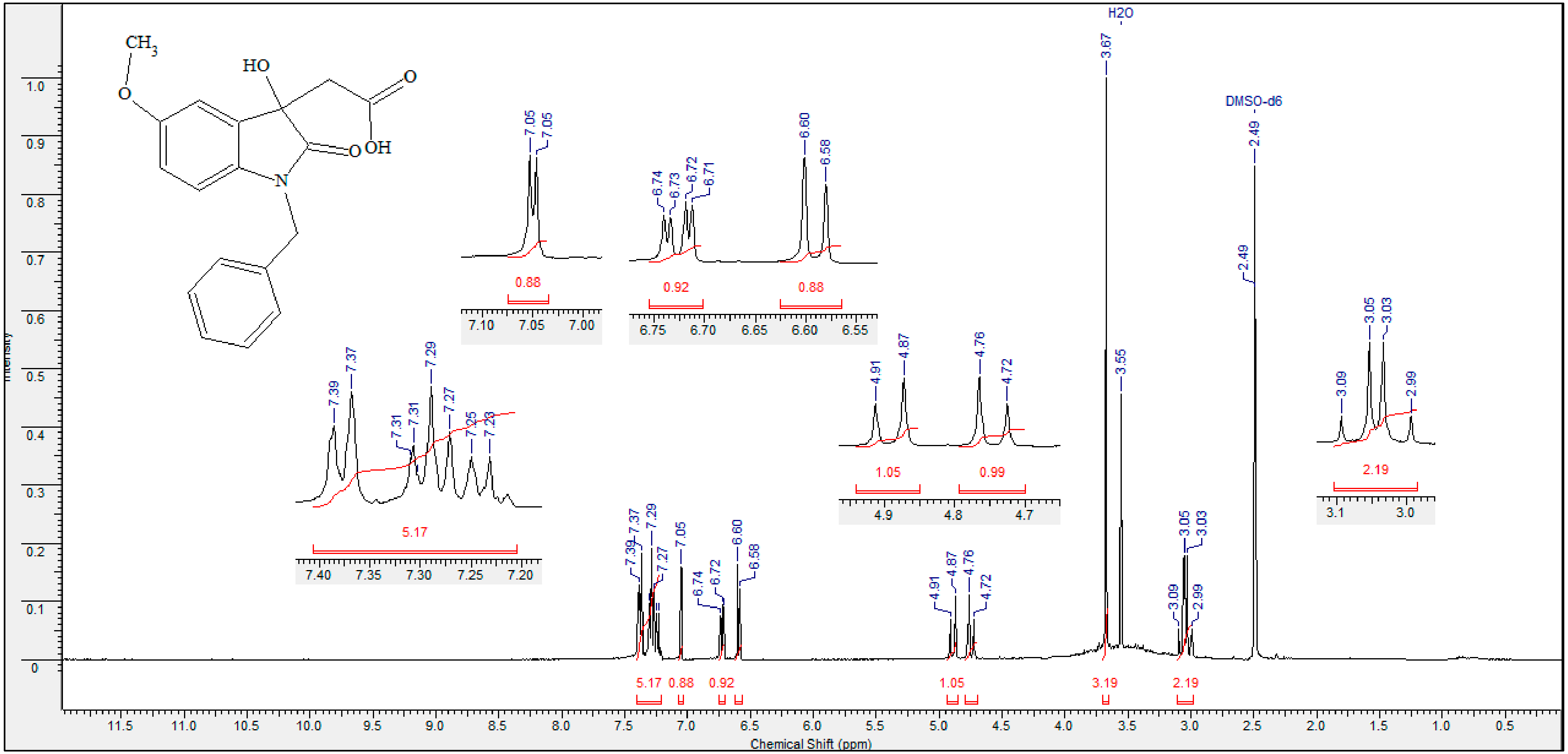

S29. 13C NMR spectrum of (1-benzyl-3-hydroxy-5-methoxy-2-oxo-2,3-dihydro-1H-indol-3-yl)acetic acid (**3i**)

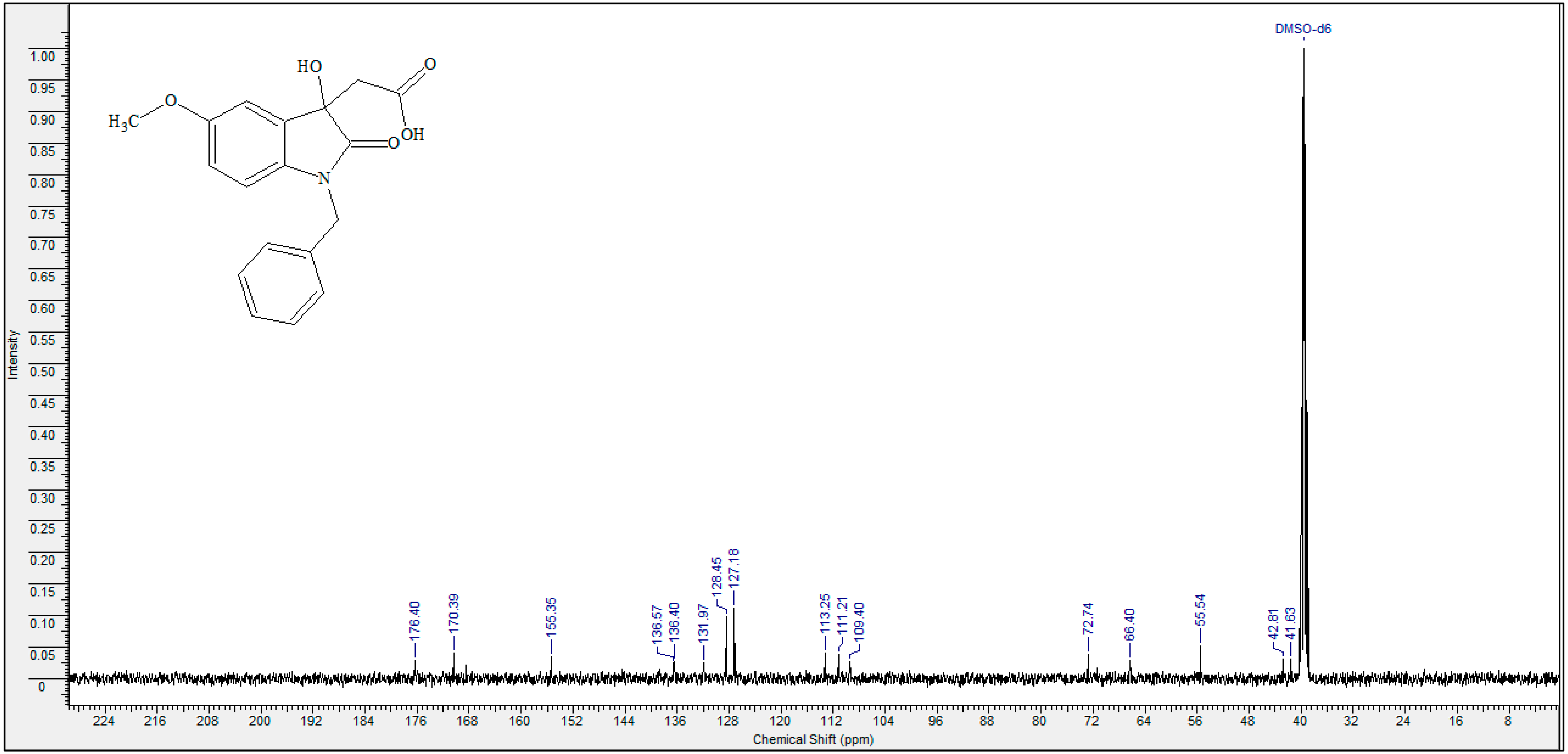

HPLC data of obtained compounds

S30 (3-hydroxy-5-nitro-2-oxo-2,3-dihydro-1H-indol-3-yl)acetonitrile (2a)

C:\Users\Dzaha...\2a_0.6mcl1.lcd Injection 1 PDA - Total Absorbance Chromatogram

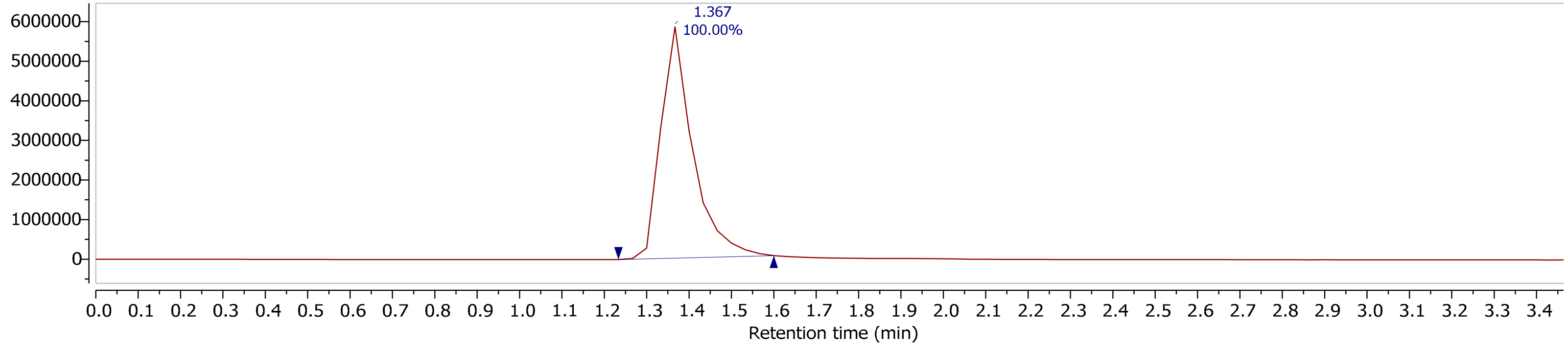

C:\Users\Dzaha...\2a_0.6mcl1.lcd Injection 1 Event 2: MS(-)...0 - 2000.00 Da) TIC

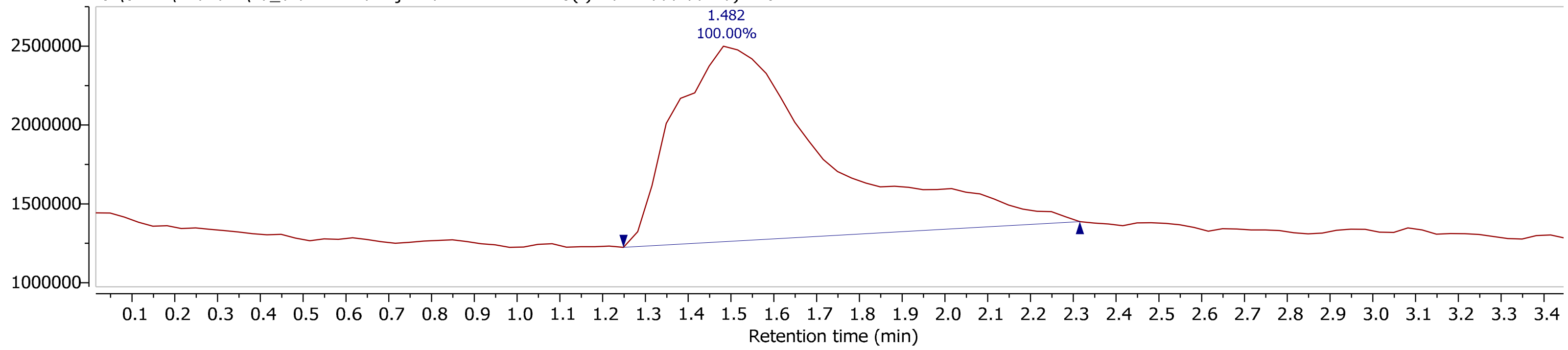

C:\Users\Dzaha...\2a_0.6mcl1.lcd Injection 1 Event 2: MS(-)...0 - 2000.00 Da) MS - spectrum 1.48

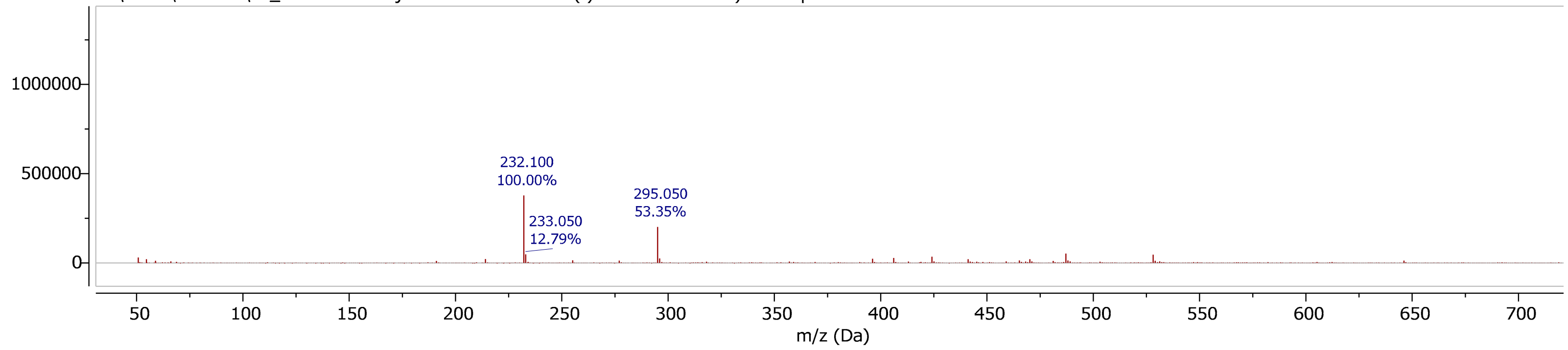

S31 (3-hydroxy-7-nitro-2-oxo-2,3-dihydro-1H-indol-3-yl)acetonitrile (2b)

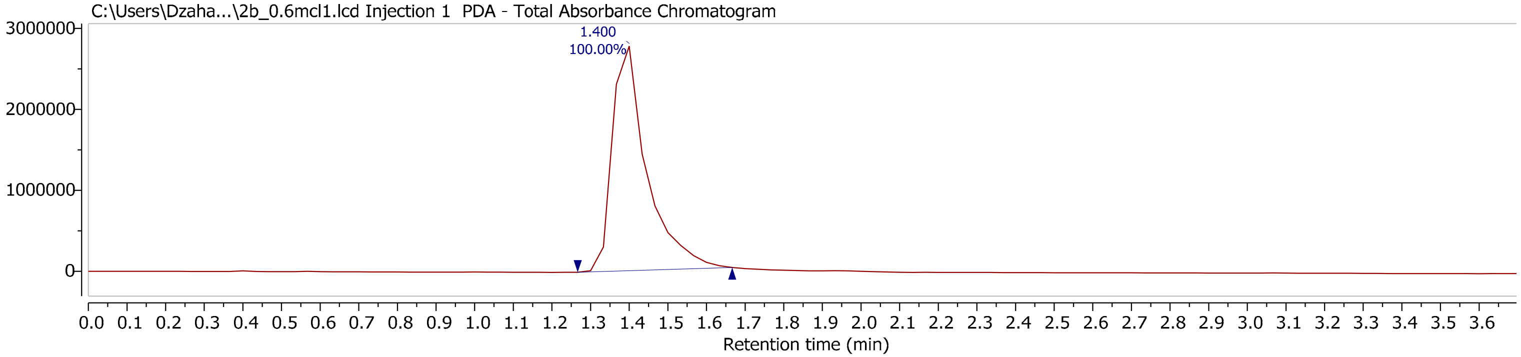

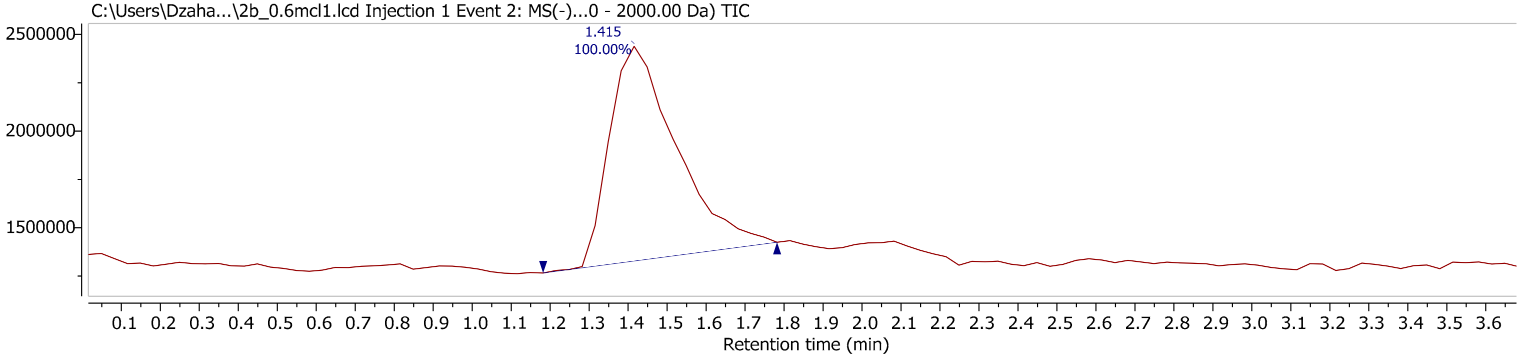

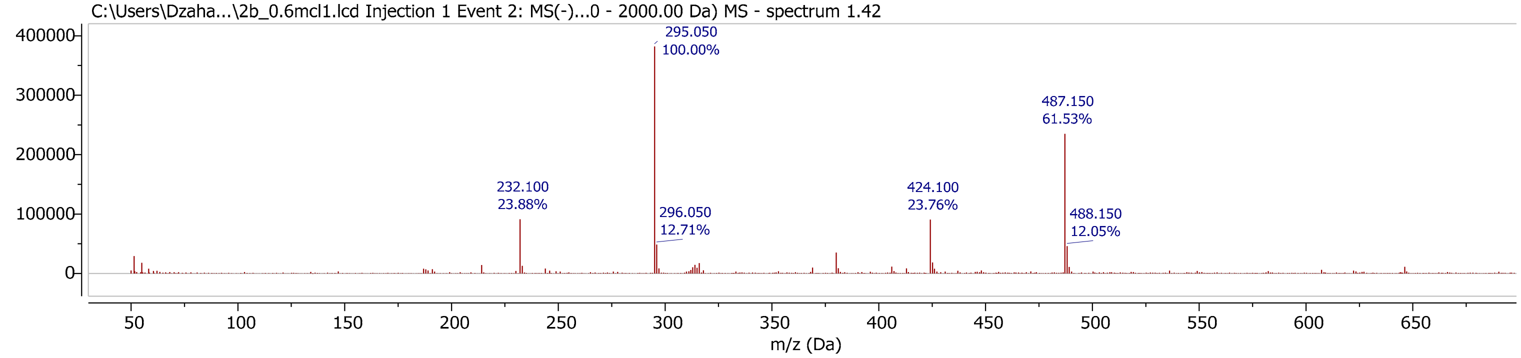

S32 (3-hydroxy-5-methoxy-7-nitro-2-oxo-2,3-dihydro-1H-indol-3-yl)acetonitrile (**2e**)

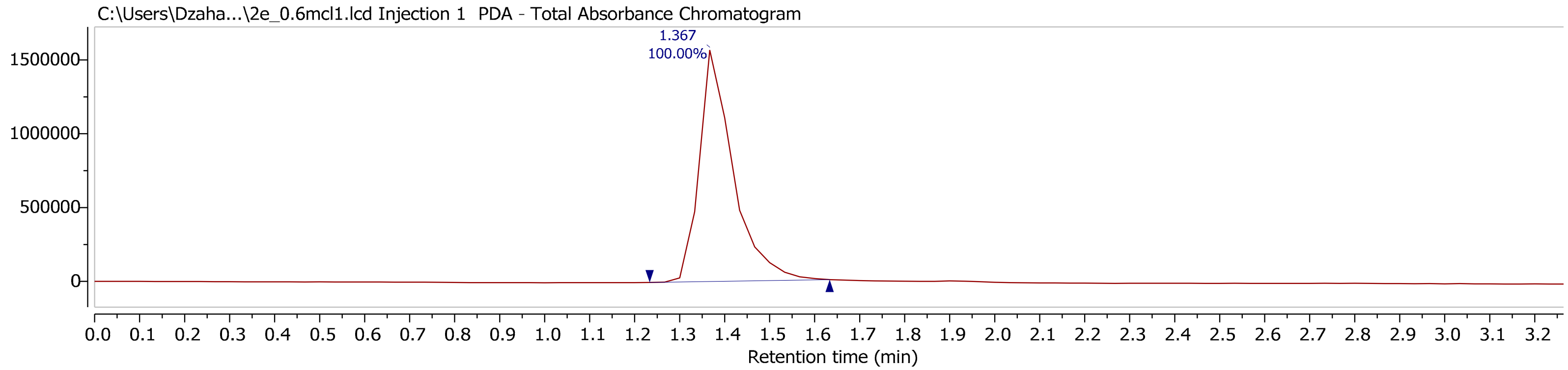

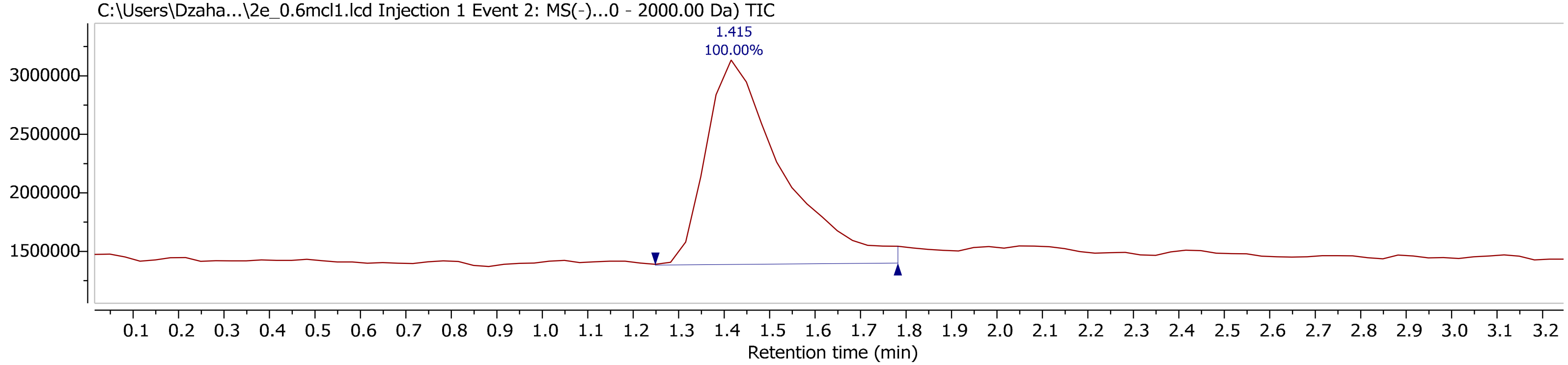

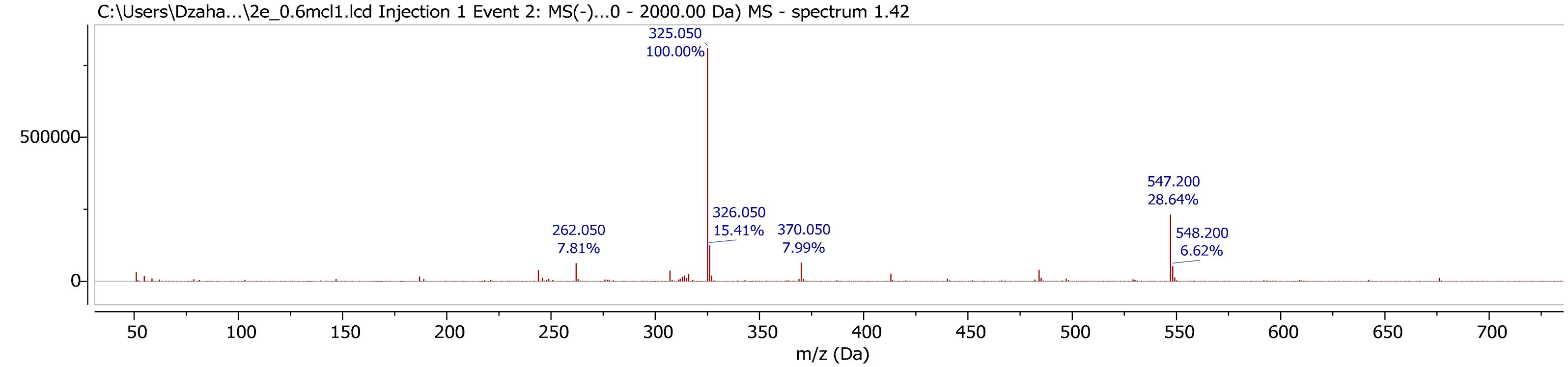

S33 (3-hydroxy-5,7-dinitro-2-oxo-2,3-dihydro-1H-indol-3-yl)acetonitrile (**2f**)

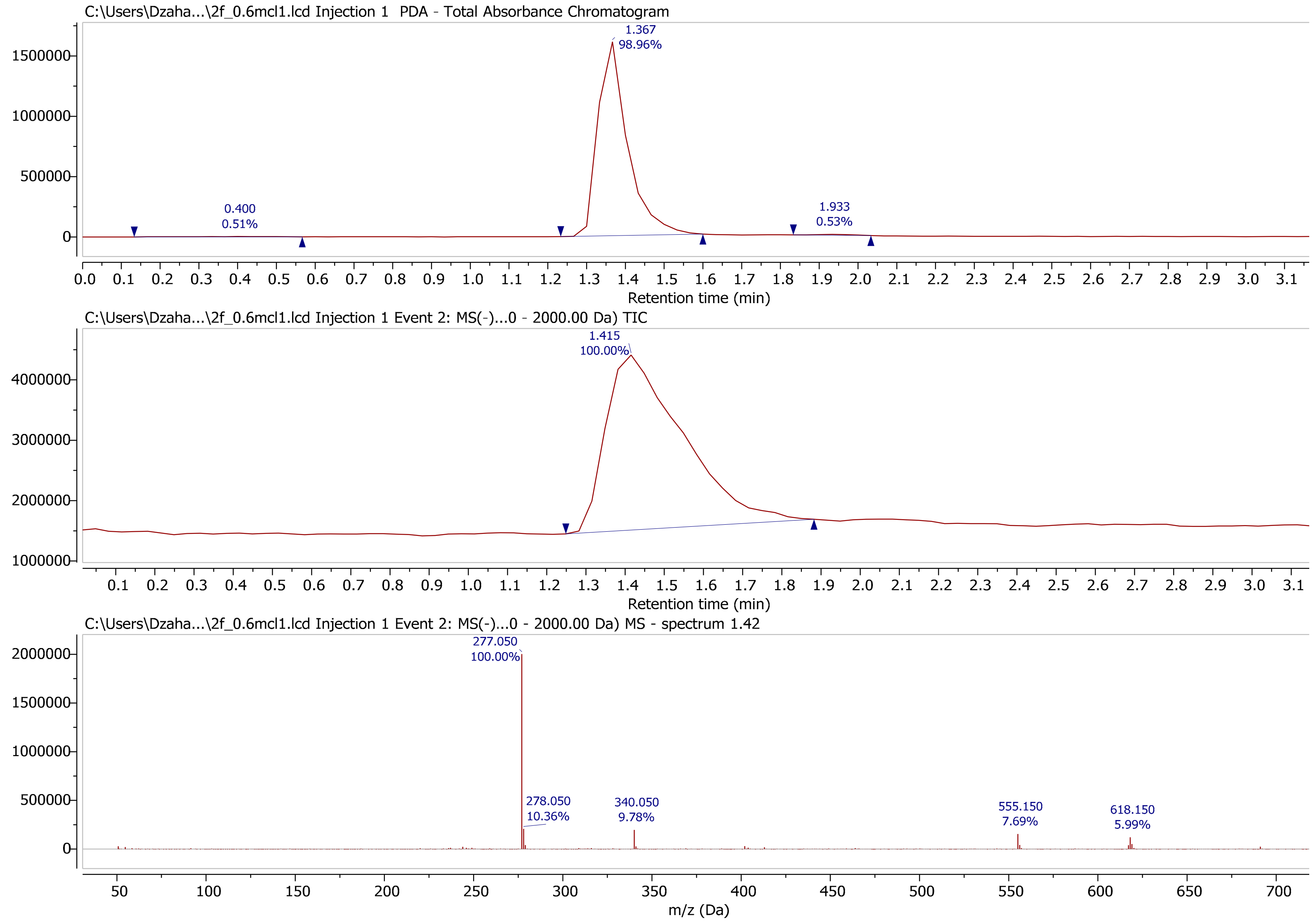

S34 (5-bromo-3-hydroxy-2-oxo-2,3-dihydro-1H-indol-3-yl)acetonitrile (**2h**)

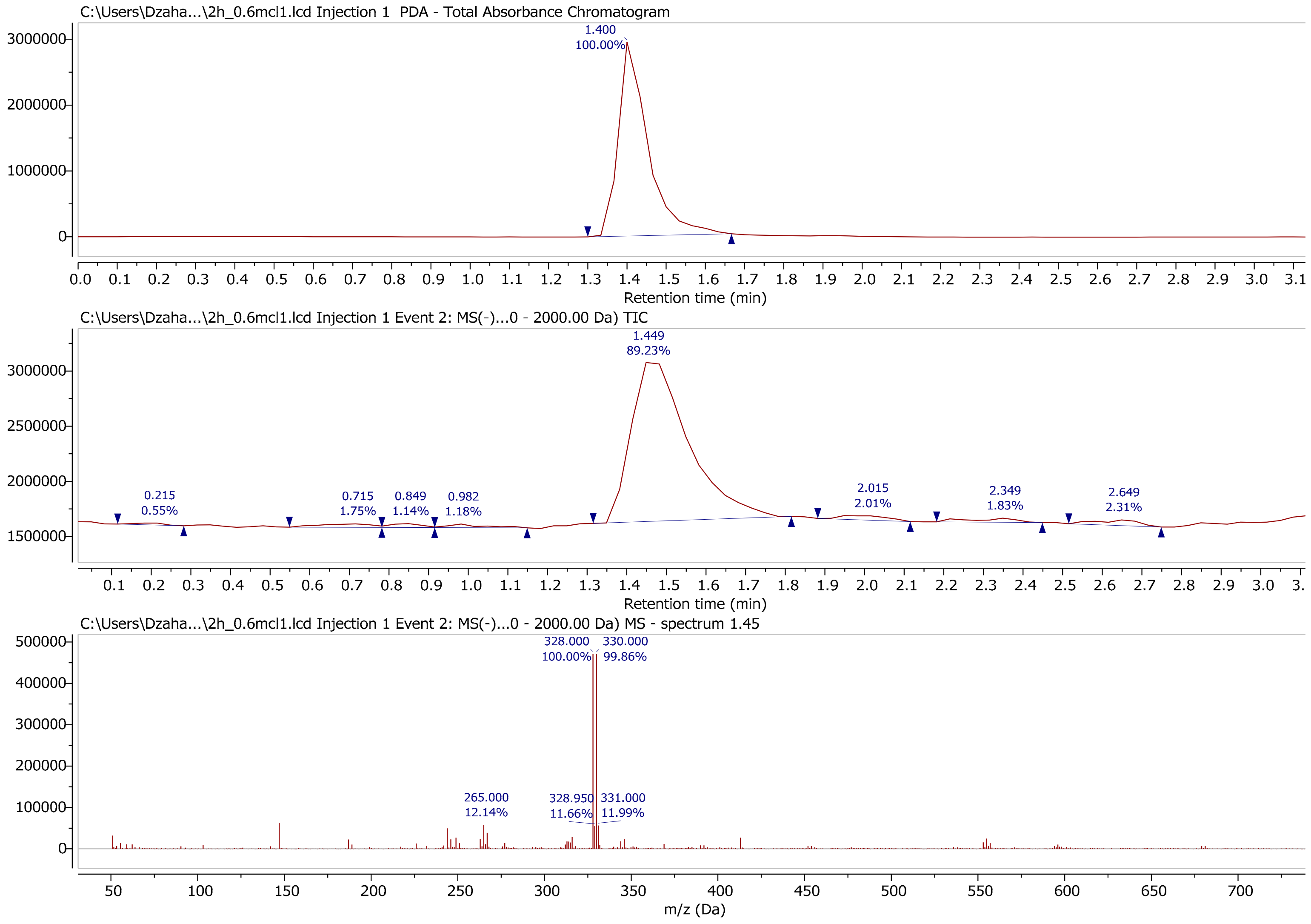

Supplement: Supplementary file 1 [file ijms-24-05101-s001.zip › ijms-2230052-supplementary.pdf]
